# Cotton *CENTRORADIALIS*/*TERMINAL FLOWER 1*/*SELF-PRUNING* genes functionally diverged to differentially impact plant architecture

**DOI:** 10.1093/jxb/ery324

**Published:** 2018-09-10

**Authors:** Sarah F Prewitt, Brian G Ayre, Roisin C McGarry

**Affiliations:** BioDiscovery Institute, Department of Biological Sciences, University of North Texas, Union Circle, Denton, TX, USA

**Keywords:** Cotton, CETS, determinate, domestication, indeterminate, patterns, plant architecture

## Abstract

Genes of the *CENTRORADIALIS*/*TERMINAL FLOWER 1*/*SELF-PRUNING* (*CETS*) family influence meristem identity by controlling the balance between indeterminate and determinate growth, thereby profoundly impacting plant architecture. Artificial selection during cotton (*Gossypium hirsutum*) domestication converted photoperiodic trees to the day-neutral shrubs widely cultivated today. To understand the regulation of cotton architecture and exploit these principles to enhance crop productivity, we characterized the *CETS* gene family from tetraploid cotton. We demonstrate that genes of the *TERMINAL FLOWER 1* (*TFL1*)-like clade show different roles in regulating growth patterns. Cotton has five *TFL1*-like genes: *SELF-PRUNING* (*GhSP*) is a single gene whereas there are two *TFL1*-like and *BROTHER OF FT* (*BFT*)-like genes, and these duplications are specific to the cotton lineage. All genes of the cotton *TFL1*-like clade delay flowering when ectopically expressed in transgenic Arabidopsis, with the strongest phenotypes failing to produce functional flowers. *GhSP*, *GhTFL1-L2*, and *GhBFT-L2* rescue the early flowering *Attfl1-14* mutant phenotype, and the encoded polypeptides interact with a cotton FD protein. Heterologous promoter::GUS fusions illustrate differences in the regulation of these genes, suggesting that genes of the *GhTFL1*-like clade may not act redundantly. Characterizations of the *GhCETS* family provide strategies for nuanced control of plant growth.

## Teaser

Gene duplications within the *TFL1*-like clade of tetraploid cotton are specific to the cotton lineage, and paralogs functionally diverged to differentially impact plant architecture.

## Introduction

Plant architecture is a fundamental determinant of crop productivity and management. Each plant’s architecture is specified by meristems that exist along a continuum from strictly indeterminate, leading to reiterative vegetative growth, to highly determinate, in which the stem cell niche is consumed in the differentiation of a terminal structure. The dynamic placement of primary meristems along this continuum accounts for monopodial or sympodial branches and inflorescences, and terminal shoots such as flowers or thorns; the position of secondary meristems (cambia) along the continuum contributes to herbaceous or woody growth; and the position of organ-specific meristems (primordia) contributes to final organ shape (Sussex and Kerk, 2001). Understanding the regulation of meristem identity can be valuable for enhancing plant architecture and improving crop yield.

Meristem identity is influenced by a wide array of genes, including members of the *CENTRORADIALIS*/*TERMINAL FLOWER 1*/*SELF-PRUNING* (*CETS*) gene family that includes the *TERMINAL FLOWER 1* (*TFL1*)-like, *FLOWERING LOCUS T* (*FT*)-like, and *MOTHER OF FT AND TFL1* (*MFT*)-like clades. *TFL1*-like genes are generally recognized as floral repressors since the Arabidopsis *tfl1* mutant and the homologous *centroradialis* from snapdragon (*Antirrhinum majus*) produce terminal flowers (Shannon and Meeks-Wagner, 1991; Bradley et al., 1996, 1997), and the tomato (*Solanum lycopersicum*) homolog *self-pruning* (*sp*) hastens termination of sympodial growth (Yeager, 1927; Pnueli *et al.*, 1998). *TFL1* is a key gene regulating developmental transitions: *AtTFL1* overexpression prolongs vegetative and reproductive phases in Arabidopsis, and inhibits floral meristem formation by delaying the up-regulation of floral meristem identity genes (Ratcliffe *et al.*, 1998; Hanzawa *et al.*, 2005; Baumann *et al.*, 2015). *AtTFL1* transcripts do not accumulate in the shoot apical meristem during the vegetative phase of growth, but strong expression is detected in the apices of young inflorescences and axillary meristems to prevent premature termination as flowers (Bradley *et al.*, 1997; Ratcliffe *et al.*, 1998, 1999; Conti and Bradley, 2007).

While AtTFL1 maintains indeterminate growth in the shoot apex, another *CETS* gene product, AtFT, terminates growth by promoting the transition from vegetative to floral meristems (Zeevaart, 2008). Although AtFT acts antagonistically to AtTFL1, the polypeptides are highly similar, and amino acid residues near the ligand binding site are critical for FT- or TFL1-like activities (Hanzawa *et al.*, 2005; Ahn *et al.*, 2006; Ho and Weigel, 2014; Wang *et al.*, 2015). *AtFT* is expressed in companion cells of the leaf vasculature and the encoded protein is the long-distance flowering signal florigen (Jaeger and Wigge, 2007). In the shoot apex, FT complexes with the transcription factor FD and 14-3-3 proteins to activate expression of floral meristem identity genes (Abe *et al.*, 2005; Wigge *et al.*, 2005; Taoka *et al.*, 2011). The functions of *FT* orthologs from other species are conserved, and similarly promote flowering and determinate growth. The dynamic balance of FT to TFL1, and their homologs in other species, is hypothesized to be responsible for establishing the determinate and indeterminate growth patterns that contributes to each plant’s architecture (Pnueli *et al.*, 1998; Lifschitz *et al.*, 2006, 2014; Shalit *et al.*, 2009).

Lineage-specific duplications of *FT*- and *TFL1*-like genes led to redundancy and neo-functionalization within species, and these events have influenced plant architecture. *TWIN SISTER OF FT* (*TSF*) in Arabidopsis acts redundantly with *FT* as a mobile flowering signal (Yamaguchi *et al.*, 2005), and *ft tsf* double-mutants are photoperiod-insensitive (Jang *et al.*, 2009). Null alleles of the *AtTFL1* paralog *arabidopsis thaliana centroradialis* (*atc*) do not have an early flowering phenotype, but ectopic expression of *ATC* delays flowering and produces extra branches, much like ectopic *TFL1* expression (Mimida *et al.*, 2001). In sugar beet (*Beta vulgaris*), poplar (*Populus* spp.), and tomato, *FT* paralogs are floral activators and repressors, and their coordinated activities are critical for seasonal cycles of flowering (Pin *et al.*, 2010; Hsu *et al.*, 2011; Soyk *et al.*, 2017). For example, BvFT1 in biennial sugar beet prevents flowering before vernalization by repressing expression of florigenic *BvFT2*; during vernalization, *BvFT1* expression decreases and plants flower in spring in response to long days (Pin *et al.*, 2010). Poplar *FT1* promotes reproductive onset in response to winter temperatures; warmer temperatures and longer days suppress *FT1* and activate *FT2* expression, which promotes vegetative growth and reproductive bud development (Hsu *et al.*, 2011). Tomato domestication involved selection for photoperiod-insensitive flowering, a trait driven by variation in the *cis*-regulation of the florigen paralog and flowering repressor *SP5G* (Soyk *et al.*, 2017). Rice (*Oryza sativa*) has 13 *FT*-like genes: *Heading date 3a* is activated by short-day photoperiods to induce flowering while *Rice flowering locus T1* promotes flowering under long days (Kojima *et al.*, 2002; Komiya *et al.*, 2008, 2009). Thus, detailed understanding of the roles of *FT*- and *TFL1*-like genes can illuminate aspects of architectural regulation in many plants.

Cotton (*Gossypium hirsutum*) is the world’s premier fiber crop and an important source of oilseed and feed. Although primarily cultivated as an annual, day-neutral row crop, cotton is naturally a short-day photoperiodic perennial. Its perennial growth habit complicates crop management strategies, and more determinate architectures are desired. While cotton varieties range from photoperiodic trees to day-neutral shrubs, branching architectures show conserved patterns: the main stem and basal branches of cotton are vegetative and monopodial, and the reproductive fruiting branches on both perennial and day-neutral varieties are sympodial. Both robust monopodial and asynchronous sympodial growth are maintained simultaneously. We have previously demonstrated that the *FT* ortholog *SINGLE FLOWER TRUSS* (*GhSFT*) and *TFL1* homolog *SELF-PRUNING* (*GhSP*) regulate cotton’s complex branching patterns (McGarry *et al.*, 2016). Virus-based over-expression and gene-silencing experiments showed that *GhSFT* stimulates determinate growth and sympodial branching but does not influence monopodial growth. *GhSP* is required to maintain all apices, and in its absence, both monopodial stem and sympodial branch meristems precociously terminate with a flower. Altering *GhSFT* and *GhSP* expression yielded highly determinate and productive cotton with reduced foliage and more synchronous fruiting. These results demonstrated that *GhSFT* and *GhSP* are important in regulating cotton architecture and suggested these could be targets for enhancing crop management and yield.

The *CETS* gene family is comprised of eight genes in diploid cotton (*G. raimondii* and *G. arboreum*) and sixteen in tetraploid *G. hirsutum* (McGarry *et al.*, 2016). The aim of this study was to analyse the cotton *CETS* gene family and explore the contributions of these gene products to cotton growth patterns. We cloned the *CETS* gene family from cotton, and characterized each member using phylogenetics, spatial and meristem-specific expression in photoperiodic and day-neutral cotton varieties grown under different photoperiods, protein–protein interactions, and transgenic analyses. We demonstrate that the *CETS* genes are differentially expressed in cotton meristems during development and under different photoperiod regimes. All genes of the *GhTFL1*-like clade impact flowering time and phase change in Arabidopsis, but functional distinctions are observed among paralogs: only the *GhSP*, *GhTFL1-L2*, and *GhBFT-L2* gene products interact with a cotton FD and rescue the Arabidopsis *tfl1-14* early flowering defect. Promoter–reporter gene analyses further emphasize regulatory distinctions among paralogs. Collectively, these findings suggest that genes of the cotton *TFL1*-like clade show distinct functions in regulating growth.

## Materials and methods

### Plant materials and growth conditions

Arabidopsis Columbia-0 (Col-0), *ft-10* (ABRC stock # CS9869), and *tfl1-14* (ABRC stock #CS6238) were germinated on soil or half-strength MS medium (PhytoTechnology Laboratories, Lenexa, KS, USA) supplemented with 1% sucrose. Seeds were stratified at 4 °C for 3 d, and transferred to chambers (Percival Scientific Inc., Perry, IA, USA) with day lengths as indicated in the text (22 /18 °C day/night) and light intensity of 120–150 µmol photons m^–2^ s^–1^ at leaf level. Growth of *G. hirsutum* accessions Texas 701 (TX701; wild, photoperiodic) and DeltaPine (DP61; domesticated, day-neutral) was as described by McGarry *et al.* (2016).

### Phylogenetic analyses

Cotton *CETS* genes were identified by tblastn searches using the six Arabidopsis CETS protein sequences (AtFT, AtTSF, AtMFT, AtTFL1, AtBFT, and ATC) as queries against *G. raimondii* (D_5_ genome, JGI assembly version 2.0, annotation version 2.1) (Paterson *et al.*, 2012), *G. arboreum* (A_2_ genome, BGI assembly version 2, annotation version 1.0) (Li *et al.*, 2014) and *G. hirsutum* (AD_1_ genome, NAU-NBI assembly version 1.1, annotation version 1.1; and Tx-JGI assembly version 1.0, annotation version 1.1) (Zhang *et al.*, 2015; Saski *et al.*, 2017) assemblies at CottonGen (www.cottongen.org;Supplementary Table S1 at *JXB* online). Predicted CETS polypeptide sequences from *G. raimondii*, *G. arboreum*, and *G. hirsutum* were aligned with CETS proteins from Arabidopsis (*A. thaliana*), tomato (*Solanum lycopersicum* and *S. pimpinellifolium*), jute (*Corchorus capsularis* and *C. olitorius*), cacao (*Theobroma cacao*), and moss (*Physcomitrella patens*) using neighbor-joining (NJ) clustering with Clustal Omega (Sievers *et al.*, 2011) (Supplementary Fig. S1). Cotton, Arabidopsis, tomato, jute, and cacao are eudicots; tomato is an asterid; cotton, Arabidopsis, jute, and cacao are rosids. Cotton, cacao, and jute are Malvaceae species, and cacao is the closest relative of cotton with a high-quality genome sequence. Default parameters for multiple sequence alignment were used as follows: matrix Gonnet, gap open 10, gap extension 0.2, gap distance 5, and clustering NJ. A phylogenetic tree based on the multiple sequence alignment was constructed using the bootstrap test by the maximum likelihood method in Mega 7 (Kumar *et al.*, 2016). The evolutionary history was inferred by using the maximum likelihood method based on the Jones–Taylor–Thornton (JTT) matrix-based model. The tree with the highest log-likelihood (–6296.89) is shown in Supplementary Fig. S2. Initial tree(s) for the heuristic search were obtained automatically by applying NJ and BioNJ algorithms to a matrix of pairwise distances estimated using a JTT model, then selecting the topology with superior log-likelihood value. The analysis involved 73 amino acid sequences. All positions with less than 80% site coverage were eliminated. That is, fewer than 20% alignment gaps, missing data, and ambiguous bases were allowed at any position. There was a total of 170 positions in the final dataset. The resultant tree was rooted with *Physcomitrella patens* sequences.

### Vector construction

Cloning was by standard molecular biology techniques (Sambrook and Russel, 2001) and yeast homologous recombination (described below). Restriction endonucleases were from New England Biolabs (Ipswich, MA, USA). Oligonucleotides were synthesized by Sigma-Aldrich (St. Louis, MO, USA). All constructs were sequenced for accuracy (Eurofins, Louisville, KY, USA).

The coding sequences of five *CETS* genes were obtained from total RNA isolated from leaves and apices of *G. hirsutum* using a hot borate procedure (Wan and Wilkins, 1994) followed by column purification (Zymo Research, Irvine, CA, USA), and reverse transcription using an oligo dT_23_ primer and Superscript III (Invitrogen, Carlsbad, CA, USA). The coding sequences of *SFT*, *BFT-L1*, and *TFL1-L2* were synthesized (GeneArt® Gene Synthesis, Invitrogen) based upon the diploid reference sequences available at the time; these encoded polypeptides share 99% amino acid identity with the proteins predicted from *G. hirsutum* annotations.

To overexpress each *GhCETS* coding sequence in Arabidopsis, sequences were PCR-amplified using Phusion polymerase and primers (Supplementary Table S2). PCR products were digested with Eco*RI* and Xba*I* and cloned into the same sites of pART7 (Gleave, 1992). Each *35S*_*pro*_*::GhCETS* expression cassette was released by Not*I* digestion, and cloned into the unique Not*I* site of binary vector pART27 (Gleave, 1992).

For yeast two-hybrid assays, the *GhCETS* coding sequences were PCR-amplified using Phusion polymerase with oligonucleotides as indicated in Supplementary Table S2. PCR products were column-purified, and digested with EcoR*I* and Sal*I* (cloning strategy for *GhSFT*, *GhSP*, *GhBFT-L1*, *GhBFT-L2*, and *GhMFT-L2*), Eco*RI* and Pst*I* (cloning strategy for *GhTFL1-L1* and *GhMFT-L1*), or Nde*I* and BamH*I* (cloning strategy for *GhTFL1-L2*), and cloned in the same sites of pGBKT7 (Clontech, Mountain View, CA, USA) to produce in-frame fusions with the *Gal4* DNA binding domain. *GrFD*, flanked by Nde*I* and BamH*I* sites, was synthesized (General Biosystems, Inc., Morrisville, NC, USA), digested with Nde*I* and BamH*I*, and cloned in the same sites of pGADT7 AD (Clontech, Mountain View, CA, USA) to produce an in-frame fusion with the *Gal4* DNA activation domain.

To generate fusions of the *GhCETS* promoter and the *E. coli* β-glucuronidase (GUS) gene (promoter::*uidA*), 2-kb promoter fragments were amplified by PCR from recombinant yeast shuttle vectors (described below) with the oligonucleotides listed in Supplementary Table S3. PCR products were column-purified, digested with Sbf*I* and Xba*I*, and cloned into the same sites of binary vector pGPTV-BAR (Becker *et al.*, 1992).

### Yeast homologous recombination and two-hybrid interaction traps

Genomic clones were constructed by yeast homologous recombination (Gibson, 2009). Each *GhCETS* promoter, coding sequence with introns, and terminator were separately PCR-amplified from *G. hirsutum* genomic DNA using Phusion and Phire polymerases and primers designed to provide 40 nucleotides of end-homology between flanking fragments (Supplementary Table S4).

In brief, a 4-system shuttle vector, pSFP100, designed for growth and selection in *E. coli*, *Saccharomyces cerevisiae*, *Agrobacterium tumefaciens*, and T-DNA transfer into plant cells, was constructed. Binary plasmid pCAMBIA0390 was linearized with Hind*III*. Yeast sequences *ARS-CEN-HIS3* were PCR-amplified from pRS313 (Sikorski and Hieter, 1989) using Phusion and Phire polymerases. To generate a plant selectable marker, the *NOS*_*pro*_*::BAR:CaMV35SpA* cassette was constructed by amplifying the *NOS* promoter from pGPTV-BAR (Becker *et al.*, 1992) and the *BAR::CaMV35SpA* from pMDC123 (Curtis and Grossniklaus, 2003) by overlapping PCR. PCR products and linearized pCAMBIA0390 were introduced into yeast cells for homologous recombination, with both cassettes inserted between the left and right borders of pCAMBIA0390. Shuttle vector pSFP100 was linearized with Bam*HI* and Eco*RI* to expose end-homology to PCR fragments (Prewitt, 2017). Linearized plasmid and column-purified PCR fragments were co-transformed into yeast strain PJ694a (James *et al.*, 1996) by standard techniques (Gietz *et al.*, 1995), and transformants selected by histidine prototrophy.

For yeast two-hybrid assays, bait and prey plasmids were co-transformed in PJ694a by standard techniques (Gietz *et al.*, 1995; James *et al.*, 1996). Transformants were plated in 10-fold serial dilutions on –Leu–Trp and on –Leu–Trp–His to detect interactions. Plates were incubated at 30 °C for 48 h, and imaged.

### Plant transformations and GUS analyses

Arabidopsis was transformed via the floral dip method (Clough and Bent, 1998), and transformants were selected using Finale herbicide (20 mg l^–1^ glufosinate-ammonium), phosphinothricin (10 mg l^–1^ glufosinate ammonium, Gold Biotechnology, St. Louis, MO), or kanamycin (100 mg l^–1^).

*GhCETS* promoter::*uidA* fusions were analysed in the T1 generation. Histochemical staining used 1 mM X-gluc (5-bromo-4-cloro-3-indolyl-β-D-glucuronic acid) in 50 mM sodium phosphate, pH 7, with 0.2 % Triton. Vacuum-infiltrated tissues were incubated at 37 °C for 24 h, and cleared with 70 % ethanol. Tissues were visualized using a SMZ1500 stereomicroscope (Nikon) with a SPOT Insight 2 CCD camera (Diagnostic Instruments Inc., Sterling Heights, MI, USA).

### *In silico* transcription factor analysis

Promoters were analysed using the regulation prediction tool at PlantRegMap (http://plantregmap.cbi.pku.edu.cn/) with the binding-site prediction threshold set at 1*e*^–5^. Putative *G. raimondii* transcription factors conserved in regulation of all orthologs were assessed for enrichment of gene ontology (GO) terms with *P*<0.01 as the threshold for significance. Enriched GO terms were visualized using REVIGO (http://revigo.irb.hr/), with the following parameters: allowed similarity, medium (0.7); database with gene ontology term size, Arabidopsis thaliana; semantic similarity measure, SimRel; *P*-values from GO enrichment analysis were provided.

### Expression analyses and RNA-seq from meristems

Spatial expression profiles were as described by McGarry *et al.* (2016) using the oligonucleotides indicated in Supplementary Table S5. For RNA-seq analysis, apices from different developmental stages of day-neutral DP61 and photoperiodic TX701 grown under different photoperiods were sampled and placed in 100% acetone. Meristems were dissected from apices, with the leaves flanking each meristem included as controls. The isolated meristems that were sampled included the following: (1) monopodial main stem from juvenile DP61; (2) monopodial main stem from juvenile TX701; (3) adult monopodial main stem from TX701 grown under long-day (vegetative) conditions (plants were not flowering); (4) monopodial lateral branches from TX701 grown under long days (plants were not flowering); (5) adult monopodial main stem from TX701 grown under inductive short days after the transition to reproductive growth (plants had flowering sympodial branches); and (6) adult sympodial fruiting branches from TX701 grown under short days (photoperiod-induced fruiting branches). Each developmental sample had three biological replicates, each comprised of four meristems. Total RNA was isolated using hot borate (Wan and Wilkins, 1994) followed by column-purification (Zymo Research, Irvine, CA, USA), and mRNA was amplified (one round) with a TargetAmp Amplification Kit (Epicenter, Madison, WI, USA). Amplified mRNA was quantified using a bioanalyser (Agilent, Santa Clara, CA, USA), and 125 ng was used to prepare Illumina TruSeq mRNA stranded libraries (Illumina, Inc., San Diego, CA, USA). A total of >30 million 50-bp single-end reads (UT Southwestern, Genomics Core facility) were obtained per library. Read quality was checked using the FastQC toolkit, and reads with low quality scores were discarded. Reads were aligned to the *G. hirsutum* reference genome (Saski *et al.*, 2017) using the Tuxedo pipeline available in the Discovery Environment at CyVerse (Bowtie v2.1.0 and TopHat v2.0.9 in conjunction with SAMtools v0.1) (Goff *et al.*, 2011). Gene FPKM (fragments per kilobase of exon model per million mapped fragments) values were calculated as normalized gene expression levels with Cufflinks v2.1.1, and Cuffdiff v2.1.1 was used to determine significant differences (Benjamini–Hochberg corrected *P*-value ≤0.05) in gene expression between samples.

## Results

### Duplications of the *TFL1*- and *BFT*- like genes are specific to cotton and absent from other malvids

The *CETS* gene family in cotton is organized in three distinct clades: *MFT*-like, *TFL1*-like, and *FT*-like. Recently released genome sequences of closely related genera enabled further inquiry into the organization of the cotton *CETS* family. Alignment of the predicted CETS polypeptides from the diploid cottons *G. raimondii* and *G. arboreum* and tetraploid *G. hirsutum* with homologs from Arabidopsis, tomato, and other Malvaceae including jute and cacao showed that *GhSFT* was the sole member of the cotton *FT*-like clade (Supplementary Figs S1, S2, Table S1). The *TFL1*-like clade had five genes in diploid cotton, with only *GhSP* lacking a paralog. *BFT*- and *TFL1*-like paralogs resulted following a duplication event specific to the cotton lineage, as they were absent from closely related cacao. The *MFT*-like clade had two genes in diploid cotton.

### *CETS* genes are differentially expressed in cotton meristems

To test whether expression of the *CETS* genes correlated with cotton architecture, we examined the spatial profiles of all gene family members in cotton varieties with different growth habits. The wild, short-day photoperiodic variety TX701 is tall with pronounced apical dominance, and leaves have five deep lobes. When grown under non-inductive long days (16/8 h), TX701 plants are vegetative; when grown under inductive short days (10/14 h), they produce sympodial branches by approximately node 20 of the main stem, and leaves subtending floral buds are lanceolate instead of lobed. The domesticated, day-neutral variety DP61 has a short, bushy growth habit, and the large leaves have three shallow lobes. DP61 produces sympodial branches by node 5 of the main stem irrespective of the day length in which it is grown, and the leaves subtending floral buds retain three shallow lobes (McGarry and Ayre, 2012; McGarry *et al.*, 2016). Both varieties maintain robust indeterminate, monopodial growth from the main stem and lower vegetative branches simultaneously with asynchronous flowering and fruiting from the upper sympodial, reproductive branches.

Using RT-qPCR, we determined the spatial expression patterns of each *CETS* gene in TX701 and DP61 grown under inductive short days (10 /14 h) and non-inductive long days (16/8 h). We have previously shown that more *GhSFT* transcript is detected in some tissues grown under short days than long days, but *GhSP* expression is not affected by day length. In general, *GhSP* expression spiked in the monopodial main stem, consistent with the strict indeterminacy of this tissue in wild-type plants (McGarry *et al.*, 2016). As with *GhSFT* and *GhSP*, *CETS* genes were weakly expressed in all tissues analysed from DP61 and TX701 (Supplementary Fig. S3). In general, *GhTFL1-L1* and *GhTFL1-L2* were expressed in the monopodial main stem, vegetative apex, and leaves of DP61 and TX701. *GhBFT-L1* was expressed in DP61 but barely detected in TX701; *GhBFT-L2* expression was not detected. *GhMFT-L1* and *GhMFT-L2* were detected at low levels in most tissues; *GhMFT-L2* expression was highest in TX701 source leaves.

Because the *CETS* genes influence meristem identity, and because *AtTFL1* is expressed dynamically in different shoot meristems (Conti and Bradley, 2007), we quantified expression in cotton meristems using RNA-seq. Meristems and the flanking leaf primordia were isolated from DP61 and TX701 seedlings, from the mature monopodial main stem of TX701 grown under long or short days, and from the apex of the branch at node 20 of TX701 grown under long or short days. As shown in Table 1, *CETS* genes were, collectively, very weakly expressed in meristems shortly after germination. Expression of *GhSFT* and *GhSP* varied significantly with developmental and environmental treatments. *GhSFT* expression was significantly up-regulated in TX701 leaf primordia flanking the branch meristem from plants grown under short days. *GhSP* expression was significantly enhanced in the branch and main stem meristems from TX701 grown under short days. *GhTFL1-L1*, *GhTFL1-L2*, *GhBFT-L1*, and *GhBFT-L2* expression were increased in the monopodial main stem meristems in long days relative to short days whereas the opposite was observed with *GhSP*. *GhTFL1-L1* and *GhTFL1-L2* expression in the branch meristem were relatively consistent between photoperiod regimes whereas expression of *GhSP* and the *GhBFT* paralogs in branch meristems were higher in short days. Expression of *GhMFT* paralogs in meristems was generally low; *GhMFT-L2* expression increased in the leaf primordia flanking the main stem meristem grown under short days, and this was similar to the spatial expression pattern. Taken together, the cotton *CETS* genes were expressed at low levels, but genes of the *TFL1*-like clade were differentially expressed in the mature main stem and branch meristems of cotton.

**Table 1.** Cotton *CETS* genes are weakly expressed in meristems and leaf primordia. Data are FPKM values from meristems isolated at different developmental stages of the day-neutral cultivated variety DP61 (D) and the wild, short-day photoperiodic TX701 (T) grown under long or short days

Gene name	Locus	DJ	DJL	TJ	TJL	TL20	TL20L	TLM	TLML	TS20	TS20L	TSM	TSML
*MFT-L1*	Gohir.D09G170700	0	0	0	0.04	0	0	0	0	0	0	0	0
	Gohir.A09G175400	0.75	0.11	0.40	0	0.90	0	1.11	0.43	0.31	0	0.54	0.11
*MFT-L2*	Gohir.D05G169400	0.05	0.03	0.06	0	0.67	0	0.80	0.07	1.73	0	0.29	6.16
	Gohir.A05G166400	0.03	0.04	0.09	0	0.61	0.05	1.81	0	1.32	0	0.50	23.10
*SFT*	Gohir.D08G248000	0	0.02	0.11	0	1.11^d^	0.81	1.66^c^	0	1.91^a^	68.48^abcd^	0.26	1.83^b^
	Gohir.A08G227700	0.61	0.05	0.05	0	0.89	0.03	0.89	0	0.87	1.08	0.64	0.12
*TFL1-L1*	Gohir.D09G135900	0.04	0.67	0.08	0.17	2.83	0.06	2.13	0.03	1.89	0	0.67	0.85
	Gohir.A09G138800	0	0.07	0	0.11	1.89	0.19	2.52	0	0.50	0	0.62	0.42
*TFL1-L2*	Gohir.D04G100700	0.26	0.19	0.52	0.27	1.29	0.10	2.05	0.19	1.85	0	0.61	0.87
*BFT-L2*	Gohir.D11G009200	0	0.07	0	0	0.33	0	1.58	0.04	1.68	0.86	0.50	1.72
	Gohir.A11G009900	0	0	0	0	0.29	0	1.42	0	0.99	0	0.08	0.21
*BFT-L1*	Gohir.D08G112000	0.02	0	0	0	0.28	0	0.91	1.00	0.73	0.29	0.39	0.35
	Gohir.A08G100900	0	0	0	0	0.24	0	1.14	0	1.09	0	0.19	0.20
*SP*	Gohir.D07G113500	0	0.09	0	0.05	3.21	0.26	1.62	0.25	5.27	0	4.71	1.11
	Gohir.A07G109700	0.02	0.02	0.11	0	2.61	0.03	0.75^ef^	0.08	10.43^e^	2.27	10.11^f^	0.05

DJ/TJ, juvenile meristems isolated at germination for DP61 (D) and TX701 (T); DJL/TJL, immature leaves flanking the DJ/TJ meristem; TL20/TS20, meristem from branch at node 20 of TX701 grown under long (L) or short (S) days ; TL20L/TS20L, immature leaves flanking the TL20/TS20 meristem; TLM/TSM, monopodial main stem meristem isolated from TX701 grown under long (L) or short (S) days ; TLML/TSML, leaves flanking the TLM/TSM meristems. Significantly different pair-wise comparisons are indicated with ^a,b^*P*<0.01, and ^c,d,e,f^*P*<0.05; *P*-values were adjusted using the Benjamini–Hoffman correction for multiple testing.

### Ectopic expression of *CETS* genes in transgenic Arabidopsis reveals subtle variations in regulating phase transitions

The *CETS* gene family is well characterized with respect to the regulated transition from vegetative to reproductive growth. To determine the roles of the cotton *CETS* genes in flowering regulation, we overexpressed each coding sequence from the *CaMV 35S* promoter in Arabidopsis Col-0 plants. The transition to reproductive growth was considered with respect to three phases: the V-phase, describing the vegetative rosette; the I1-phase, with the inflorescence bearing cauline leaves subtending axillary branches; and the I2-phase, describing an inflorescence bearing flowers (Ratcliffe *et al.*, 1998). *AtTFL1* overexpression was characterized by the I1* phase intermediate to I1 and I2, in which the axillary shoots are not subtended by cauline leaves, and shoots become more flower-like at the apex, with clusters of flowers surrounded by leaf-like organs (Ratcliffe *et al.*, 1998).

**Fig. 1.**
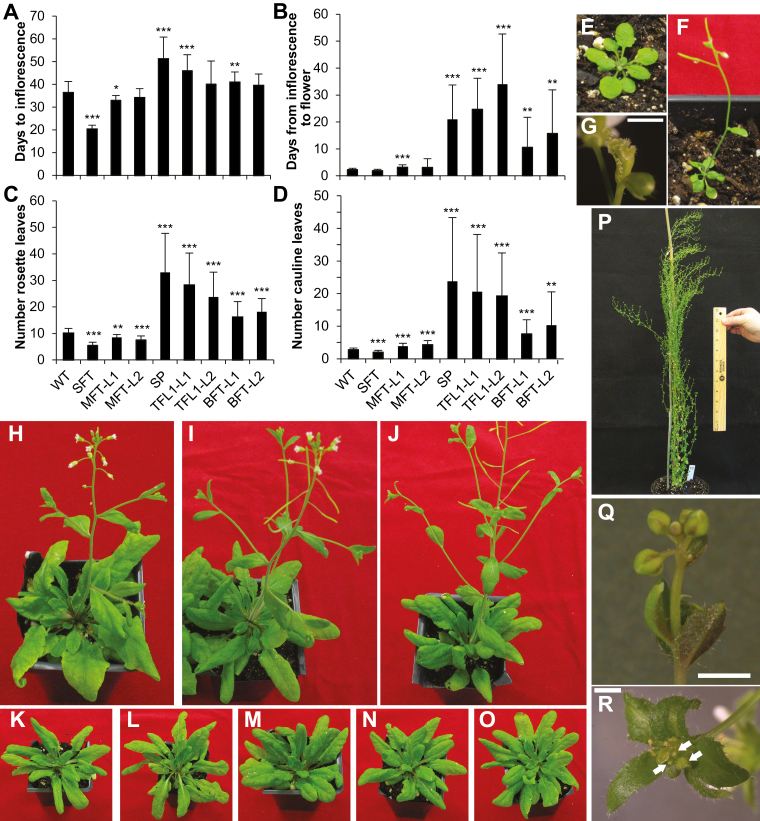
Ectopic expression of *GhCETS* genes differentially impacts on phase changes in Arabidopsis. The effects of *35S*_*pro*_*::GhCETS* upon the transition to reproductive growth were quantified using (A) the days to inflorescence, when the inflorescence measured 1 cm in height, (B) the days from inflorescence to flowering, when white petals were visible, (C) the number of rosette leaves present at flowering, and (D) the number of cauline leaves present by anthesis. Data are means (±SD) (*n*=18). Means from each *35S*_*pro*_*::GhCETS* treatment were compared to untransformed wild-type Col-0 (WT) using Student’s *t*-test: *P<0.05, **P<0.01,***P<0.001. (E) WT at 3 weeks post-germination produces a small rosette, but (F) *35S*_*pro*_*::GhSFT* lines are flowering at 3 weeks and (G) some produce homeotic terminal flowers (five out of 18 lines). (H) At 7 weeks post-germination WT plants are flowering, as are (I) *35S*_*pro*_*::GhMFT-L1* and (J) *35S*_*pro*_*::GhMFT-L2* lines. At 7 weeks, the transition to reproductive growth is delayed among (K) *35S*_*pro*_*::GhSP*, (L) *35S*_*pro*_*::GhTFL1-L1*, (M) *35S*_*pro*_*::GhTFL1-L2*, (N) *35S*_*pro*_*::GhBFT-L1*, and (O) *35S*_*pro*_*::GhBFT-L2* lines. Ectopic expression of genes of the *TFL1*-like clade produced the I1* phase. In (P), the *35S*_*pro*_*::GhSP* line remains in the I1* phase at 16 weeks post-germination. (Q) A cluster of flowers surrounded by whorled, leaf-like organs is observed in *35S*_*pro*_*::GhTFL1-L1* lines in the I1* phase. (R) A floral structure from the *35S*_*pro*_*::GhTFL1-L2* line in the I1* phase, with multiple floral buds (arrows) initiating within the innermost whorl. Scale bars are 1 mm.

Wild-type Arabidopsis grown under 12/12 h light/dark transitioned to reproductive growth by 36 d post-germination (dpg): vegetative growth consisted of 10 rosette leaves, and inflorescences had three cauline leaves (Fig. 1A–D, F, H). As expected, ectopic expression of *GhSFT* accelerated the transition to reproductive growth, with inflorescences evident by 20 dpg on plants with five rosette leaves; two cauline leaves developed on the inflorescences (Fig. 1A–E). Five out of 18 independent *35S*_*pro*_*::GhSFT* lines produced homeotic terminal flowers lacking sepals, petals, and stamens, and harboring three unfused carpels surrounding an inner fused carpel (Fig 1G); flowers preceding the terminus were normal. The Arabidopsis inflorescence is normally monopodial and indeterminate; over-expression of *GhSFT* converted the indeterminate apical meristem to a terminal floral meristem.

Overexpressing *GhSP*, *GhTFL1-L1*, *GhTFL1-L2*, *GhBFT-L1* and *GhBFT-L2* promoted indeterminate growth by extending the V- and I1-phases (Fig. 1). *35S*_*pro*_*::GhSP*, *35S*_*pro*_*:GhTFL1-L1* and *35S*_*pro*_*:GhTFL1-L2* produced 2- 3x more rosette leaves than WT; *35S*_*pro*_*::GhBFT-L1*, and *35S*_*pro*_*::GhBFT-L2* produced ~1.5× more rosette leaves compared to the wild-type (WT; Fig. 1C). Overexpression of genes from the *TFL1*-like clade produced the intermediate I1* phase: nodes became progressively more flower-like (*GhSP* is pictured in Fig. 1P; *GhBFT-L1* is pictured in Supplementary Fig. S4), with floral clusters surrounded by whorled leaf-like structures at the uppermost nodes (*GhTFL1-L1* is pictured in Fig. 1Q). At times, these structures produced abnormal flowers with floral buds originating from unfused carpels in the inner whorl (*GhTFL1-L2* is pictured in Fig. 1R). Furthermore, independent *35S*_*pro*_*::GhSP*, *35S*_*pro*_*::GhTFL1-L1*, *35S*_*pro*_*::GhTFL1-L2*, and *35S*_*pro*_*::GhBFT-L2* lines were still in the I1*-phase by 120 dpg (Fig. 1P). These findings, consistent with reports of *AtTFL1* overexpression in Arabidopsis (Ratcliffe *et al.*, 1998), indicated that all members of the *TFL1*-like clade could function as indeterminate factors, with some strongly promoting indeterminate growth and others with weaker activity.

The V-phase was slightly accelerated in the *35S*_*pro*_*::GhMFT-L1* and *35S*_*pro*_*::GhMFT-L2* lines, consistent with reports of *AtMFT* overexpression (Yoo *et al.*, 2004). Overexpression of *GhMFT*-like genes slightly extended the I1-phase, with T1 lines having four cauline leaves compared to three in the WT (Fig. 1).

### *GhSFT* and *GhTFL1*-like genes rescue Arabidopsis flowering mutants

To determine whether the cotton *CETS* gene products shared conserved functions in flowering, we introduced each gene with native regulatory sequences to Arabidopsis flowering mutants. Genomic clones comprised of 2 kb of sequence upstream of the ATG, coding sequence with introns, and 1 kb of sequence downstream of the stop codon were introduced to the late-flowering *ft-10* and early-flowering *tfl1-14* mutants.

**Fig. 2.**
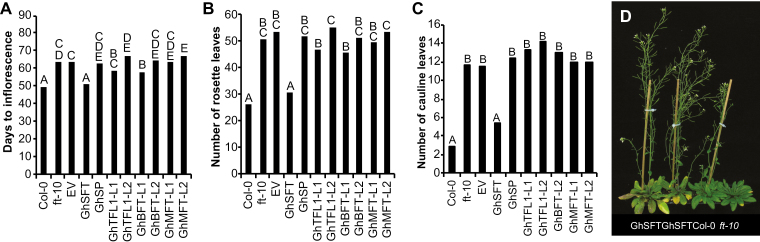
Of the cotton *CETS*, only *GhSFT*, expressed from native regulatory sequences, rescues the Arabidopsis *ft-10* flowering defect. The *GhCETS* genomic clones were introduced into the *Atft-10* mutant background. T1 lines were analysed for the ability to rescue the *ft-10* flowering defect, as quantified by (A) the days to inflorescence, (B) the number of rosette leaves formed at flowering, and (C) the number of cauline leaves present at flowering. Significant differences among genotypes (*n*=11–13 independent T1 lines; for *ft-10*, *n*=20) were determined by ANOVA, with mean separation by Tukey’s HSD test (*P*<0.05). (D) Two independent *GhSFT*_*pro*_*::GhSFT Atft-10* lines are shown with wild-type Col-0 and *ft-10* at 60 d post-germination.

Loss-of-function *ft-10* is severely delayed in determinate growth under long-day conditions, flowering after >40 rosette leaves, and produces a ‘bushy’ phenotype resulting from many inflorescence branches (Yoo *et al.*, 2005). When plants were grown in 12/12 h light/dark conditions, *ft-10* and the empty vector transformation controls flowered ~15 d later than the WT, with each producing 2× and 6× the number of rosette and cauline leaves, respectively, compared to the WT (Fig. 2). Independent T_1_*GhSFT*_*pro*_*::GhSFT* lines fully rescued the *ft-10* mutant, having similar numbers of rosette and cauline leaves and producing inflorescences within 1 d of the WT. These findings demonstrated that *GhSFT* promoted determinate growth to levels comparable to AtFT. *GhSFT* is the only cotton gene in the *FT*-like clade of the *CETS* family, and, as expected, the remaining seven cotton *CETS* did not rescue the *ft-10* mutant defect (Fig. 2).

**Fig. 3.**
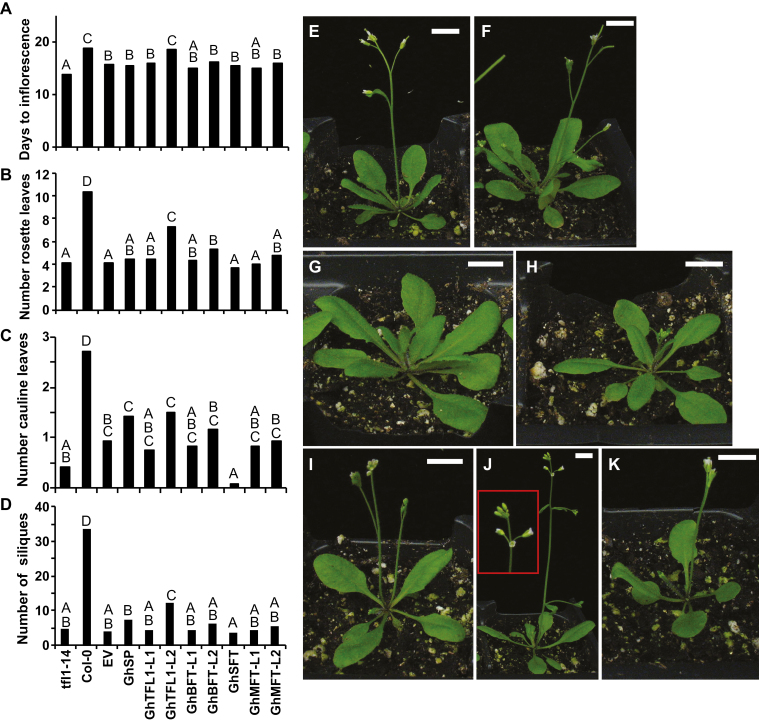
Expressing *GhSP*, *GhTFL1-L2*, or *GhBFT-L2* under native regulation partly rescues the Arabidopsis *tfl1-14* flowering defect. Genomic *GhCETS* clones were introduced into the *Attfl1-14* early-flowering mutant. T1 lines of each genotype (*n*=12) were analysed for rescue of the mutant phenotype, as quantified by (A) days to inflorescence, (B) number of rosette leaves at flowering, (C) number of cauline leaves at flowering, and (D) number of siliques. Significant differences among genotypes were determined by ANOVA, with mean separation by Tukey’s HSD test (*P*<0.05). (E) *Attfl1-14* and (F) the empty vector control in the *tfl1-14* background flower earlier than (G) wild-type Col-0. (H) *GhTFL1-L2*_*pro*_*::GhTFL1-L2 tfl1-14* lines restore the early-flowering defect to resemble the wild-type. (I) *GhBFT-L2*_*pro*_*::GhBFT-L2 tfl1-14* shows partial rescue of the early-flowering mutant. (J) *GhSP*_*pro*_*::GhSP tfl1-14* plants produce an indeterminate inflorescence (inset). (K) *GhSFT*_*pro*_*::GhSFT tfl1-14* enhances the mutant phenotype by accelerating the transition to reproductive growth; inflorescences lack cauline leaves. Images of representative lines are shown at 18 d post-germination. Scale bars are 1 cm.

A single-nucleotide substitution in the first exon of *AtTFL1* produces the early-flowering phenotype of *tfl1-14* (Schultz and Haughn, 1993). Under long days (16/8 h), *tfl1-14* flowers early: the main inflorescence terminates with a single flower or cluster of flowers, releasing the rosette axillary meristems from apical dominance, and the whorls of the terminal flower may be missing or mosaic, as described by Shannon and Meeks-Wagner (1991). Empty vector controls in the *tfl1-14* background produced terminal flowers but flowered later than *tfl1-14* plants (Fig. 3A–C, F), probably because transgenic controls were grown under selection, resulting in a general developmental delay. As anticipated, *GhSFT*_*pro*_*::GhSFT tfl1-14* accelerated flowering: the main inflorescence terminated with a flower and produced fewer cauline leaves and siliques compared to *tfl1-14* (Fig. 3C, D, K). *GhTFL1-L2*, *GhBFT-L2*, and *GhSP* rescued the *tfl1-14* phenotype to different extents. *GhTFL1-L2*_*pro*_*::GhTFL1-L2* rescued the mutant phenotype to nearly wild-type levels, flowering later and producing twice as many rosette and cauline leaves compared with *tfl1-14* or the empty vector controls (Fig. 3A–C, G, H). The inflorescence apical meristem of *GhTFL1-L2*_*pro*_*::GhTFL1-L2 tfl1-14* remained indeterminate longer than *tfl1-14* or the empty vector controls, and yielded 2.5× more siliques before termination (Fig. 3D). *GhBFT-L2*_*pro*_*::GhBFT-L2 tfl1-14* produced more rosette leaves and siliques before termination compared with controls (Fig. 3A–D, I). Similarly, *GhSP* partly rescued the flowering mutant, with the inflorescence remaining indeterminate for significantly longer, and plants producing twice as many siliques before termination (Fig. 3D, H). The remaining cotton *TFL1*-like genes failed to rescue the early-flowering phenotype (Fig. 3).

### Cotton CETS proteins interact with a cotton FD-like protein

**Fig. 4.**
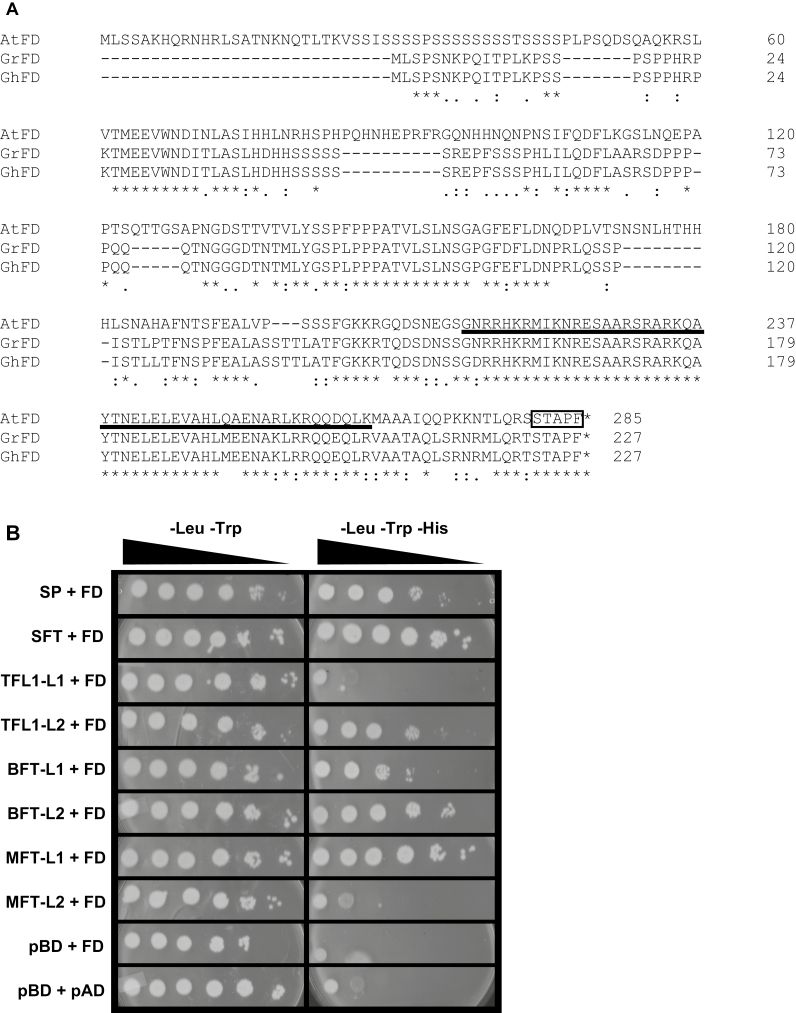
Cotton CETS proteins interact with a cotton FD. (A) FD homologs in cotton were identified using the Arabidopsis FD protein to query the *G. raimondii* and *G. hirsutum* genome sequences by tblastn. The predicted polypeptides were aligned with AtFD; the bZIP domain is underlined, and the SAP motif is boxed. (B) Bait and prey plasmids were co-transformed into yeast, and plated in 10-fold serial dilutions on medium lacking leucine and tryptophan, and on medium lacking leucine, tryptophan, and histidine.

FT interacts with 14-3-3 proteins and the bZIP transcription factor FD in the meristem to activate expression of floral meristem identity genes (Abe *et al.*, 2005; Wigge *et al.*, 2005; Taoka *et al.*, 2011). AtTFL1 similarly interacts with AtFD, preventing trans-activation of downstream genes (Hanano and Goto, 2011). To characterize the cotton CETS activities *in vivo*, we tested the interactions of the proteins with a cotton FD using yeast two-hybrid interaction screens (Fields and Song, 1989). GrFD was originally identified from the *G. raimondii* assembly, and the cDNA used. GhFD was identified in subsequent genome annotations. GrFD and GhFD had 98% identical amino acids, and both shared the bZIP and SAP domains characterizing AtFD (Fig. 4A). Each *CETS* coding sequence was fused in-frame to the DNA binding domain of the *Gal4* coding sequence, the *GrFD* coding sequence was fused in-frame to the activation domain of *Gal4*, and constructs were co-transformed into yeast cells. GhSFT and GhSP both interacted with GrFD to confer histidine prototrophy in yeast two-hybrid assays (Fig. 4B). These findings support the model for regulation of indeterminate to determinate growth through competitive binding of SP and SFT to the shared ligand. In addition, GhMFT-L1, GhBFT-L2, and GhTFL1-L2 interacted with GrFD to confer histidine prototrophy to yeast cells. That GhBFT-L2 and GhTFL1-L2 bound GrFD, in conjunction with each belonging to the TFL1-like clade, was consistent with their partial rescue of the *tfl1-14* defect. GhMFT-L1 bound FD in yeast cells, but a role for this complex in controlling plant architecture is unknown.

### *cis*- and *trans*-acting factors differentially regulate spatial and temporal expression of *CETS* genes

To better appreciate the function of *CETS* genes in plant architecture, we considered *cis*- and *trans*-regulation of their specific expression patterns over the course of development. We analysed the *CETS* promoters by fusing ~2 kb of sequence upstream from the start codon of each gene to the *uidA* (GUS) reporter, and introduced each promoter–reporter fusion to Arabidopsis Col-0. This approach was supported by evidence that regulatory cascades can be highly conserved across eudicots (Ayre *et al.*, 2003). T_1_ plants were assayed for GUS activity at three developmental time-points: young rosette (10 dpg), older rosette (20–30 dpg), and flowering plants (45–65 dpg).

**Fig. 5.**
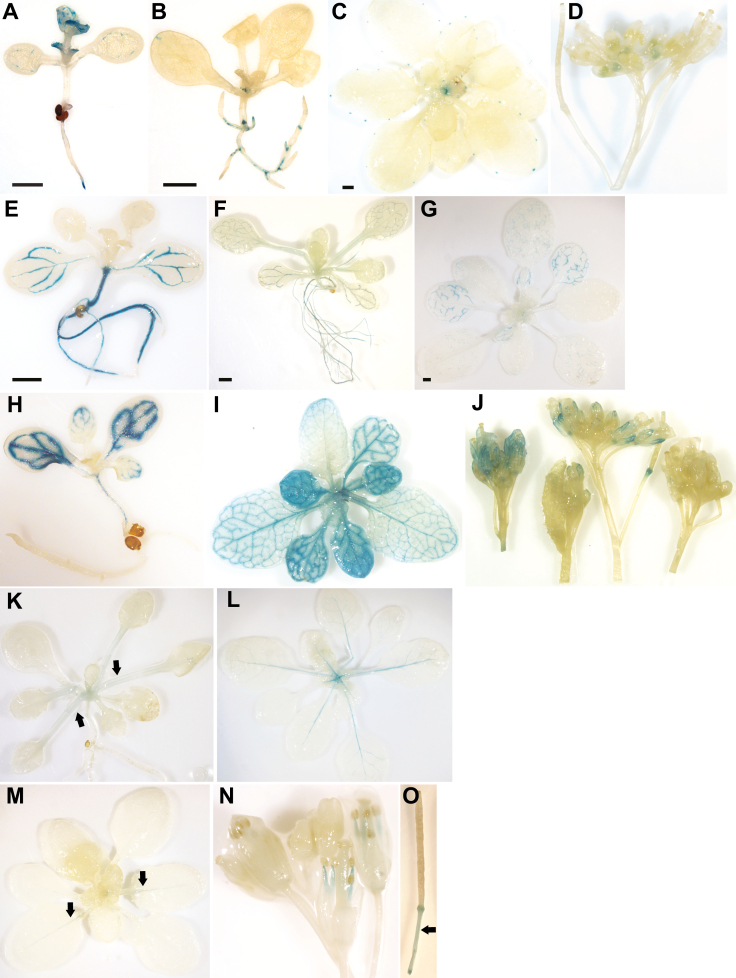
*cis*-Regulation of *CETS* expression during development. Approximately 2 kb of sequence upstream of the start codon of each *CETS* gene was cloned and used to drive *uidA* (GUS) expression. GUS staining was visualized in young rosettes [≤10 d post-germination (dpg), A, B, E, F, H, K), older rosettes (20–30 dpg, C, G, I, L, M), and flowering plants (45–65 dpg, D, J, N, O). (A) At the young rosette stage, *GhSFT*_*pro*_*::uidA* T1 lines exhibited GUS staining in the root apical meristem (observed in 16 of 28 independent lines) and in the leaf vasculature (8 of 28 T1 lines). No GUS staining was detected at later developmental stages (0 out of 12 independent lines examined at the older rosette stage and 24 independent T1 lines examined at flowering). (B–D) Among *GhSP*_*pro*_*::uidA* transgenic lines, GUS staining was limited to meristems in 20 of 32 independent lines (B) and 11 of 12 independent T1 lines (C), and in floral buds in 5 of 8 independent lines (D). (E) GUS staining in *GhTFL1-L1*_*pro*_*::uidA* plants was restricted to the vasculature in young rosettes (22 of 28 independent lines). GUS staining was not detected in older rosettes (0 out of 12 lines) or flowering plants (8 lines). (F, G) Among *GhTFL1-L2*_*pro*_*::uidA* transgenics, vascular GUS staining patterns were observed in young rosettes in 11 of 14 independent lines (F) and in older rosettes in 8 of 15 independent lines (G), but not observed in flowering plants (0 out of 16 lines). (H–J) Vascular GUS staining patterns were observed in young rosettes in 24 of 28 independent lines (H) and older rosettes in 24 of 28 lines (I), and in the sepals of flowering *GhBFT-L2*_*pro*_*::uidA* plants in 22 of 24 lines (J). (K, L) *GhMFT-L1*_*pro*_*::uidA* transgenics produced weak GUS staining in petioles in 14 of 16 independent lines (K) and in mid-ribs of rosette leaves in 14 of 16 independent lines (L). (M–O) *GhMFT-L2*_*pro*_*::uidA* plants produced weak GUS staining in petioles at the older rosette stage in 9 of 16 independent lines (M), filaments in 6 of 16 T1 lines (N), and peduncles of siliques in 9 of 16 independent lines(O). GUS staining was not observed in *GhBFT-L1*_*pro*_*::uidA* transgenics at any developmental stage (0 out of 16 lines examined at the young rosette stage, 0 of 10 lines examined at the older rosette stage, and 0 of 16 independent lines studied at flowering). Arrows indicate weak GUS staining in petioles (K), mid-ribs (M), and peduncles (O). Scale bars are 1 mm.

In young rosettes, *GhSFT*_*pro*_*::uidA* T_1_ plants demonstrated GUS activity in root apical meristems and in the distal minor veins of expanding leaves (Fig. 5A). GUS activity specifically in the minor veins is consistent with the long-distance transport described for AtFT (Wigge, 2011). GUS was not detected in older rosettes or flowering plants (roots were not examined in older plants). This finding was unexpected: we anticipated vascular expression to continue throughout development. The *GhSFT*_*pro*_*::uidA* expression pattern suggested that additional regulatory sequences may be required following the transition to reproductive growth.

The *GhSP*_*pro*_*::uidA* lines demonstrated robust GUS activity in shoot apical, shoot axillary, leaf lateral, root apical, and root lateral meristems in young rosettes, and in aerial meristems of older rosettes (Fig. 5B, C). In flowering plants, the *GhSP*_*pro*_*::uidA* lines showed GUS staining in immature floral buds, but not in flowers and siliques (Fig. 5D). These patterns of *GhSP* expression were consistent with a role in maintaining indeterminate meristems.

GUS staining was restricted to the vasculature in *GhTFL1-L1*_*pro*_*::uidA*, *GhTFL1-L2*_*pro*_*::uidA*, and *GhBFT-L2*_*pro*_*::uidA* young rosettes (Fig. 5E, F, H). Leaf vascular staining patterns resembled the source-to-sink transition, wherein X-gluc staining was observed in all veins of expanded leaves, in minor veins at the apex of expanding leaves, and was absent from unexpanded sink leaves. This staining pattern varied later in development: the source-to-sink transition pattern was observed in some *GhTFL1-L2*_*pro*_*::uidA* older rosettes (Fig. 5G), but not in flowering *GhTFL1-L2*_*pro*_*::uidA*, or in *GhTFL1-L1*_*pro*_*::uidA* older rosettes or flowering plants. In contrast, *GhBFT-L2*_*pro*_*::uidA* plants demonstrated vascular staining in older rosettes and flowering plants (Fig. 5I) and in the sepals at anthesis (Fig. 5J). Strong GUS staining was also detected in the vasculature of the root and hypocotyl in young rosettes of *GhTFL1-L1*_*pro*_*::uidA* and *GhTFL1-L2*_*pro*_*::uidA* plants (Fig. 5E, F), but not in *GhBFT-L2*_*pro*_*::uidA* plants (Fig. 5H). These exclusive vascular-staining patterns were unexpected and strikingly different from those of *GhSP* (Fig. 5B–D). No GUS activity was detected in the *GhBFT-L1*_*pro*_*::uidA* lines at any developmental stage. Taken together, these findings suggested differential regulation between paralogous genes, and among *GhTFL1*, *GhBFT*, and *GhSP*.

The *GhMFT-L1*_*pro*_*::uidA* and *GhMFT-L2*_*pro*_*::uidA* lines demonstrated weak GUS staining in petioles and mid-ribs of expanded leaves from young and mature rosettes, and in the vasculature of filaments from flowers (Fig. 5K–O).

**Fig. 6.**
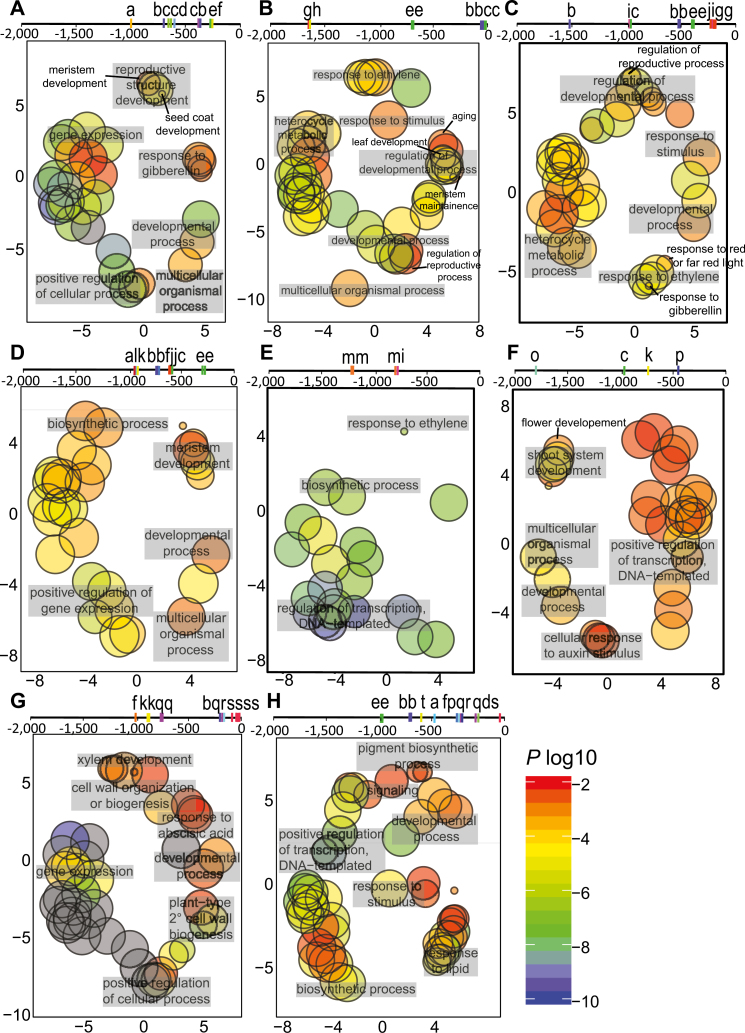
*trans*-Regulation of the cotton *CETS* genes predicts different transcription factor binding sites. Approximately 2.0 kb of promoter sequences of orthologous cotton *CETS* genes from four *Gossypium* genome assemblies were analysed for conserved regulation using the Regulation Prediction tool at PlantRegMap. Sets of *G. raimondii* transcription factors conserved in regulation were analysed for enrichment of gene ontology (GO) terms. The predicted transcription factor binding sites are shown as line diagrams above scatterplots for (A) *SFT*, (B) *SP*, (C) *TFL1-L1*, (D) *TFL1-L2*, (E) *BFT-L1*, (F) *BFT-L2*, (G) *MFT-L1*, and (H) *MFT-L2*. Along the line diagrams are the following predicted transcription factors: a, WOX; b, DOF; c, MIKC-MADS; d, trihelix; e, TCP; f, MYB/G2-like; g, BBR-BPC; h, ARR-B; i, GRAS; j, AP2; k, NAC; l, NIN-like; m, ERF; n, TALE; o, ARF; p, BES1; q, bZIP; r, bHLH; s, C2H2; and t, HD-ZIP. Each scatterplot shows cluster representations of GO terms: circle size represents the GO term generality with larger circles corresponding to general terms; circle color is based upon the *q*-values from the Fisher’s exact test used in gene enrichment analysis.

To further examine the regulation of these expression patterns, we identified predicted transcription factor binding sites within the promoters of each *CETS* gene. The orthologous *CETS* promoters from diploid *G. raimondii* (D genome) and *G. arboreum* (A genome), and tetraploid *G. hirsutum* (A_t_ and D_t_ subgenomes) were analysed for conserved regulation using the PlantRegMap software (Jin *et al.*, 2015, 2017) (Supplementary Table S6). Predicted transcription factor binding sites were analysed for enrichment of GO terms using REVIGO (Supek *et al.*, 2011; Jin *et al.*, 2017). *Trans*-acting factors predicted to regulate expression of *GhSP*, *GhTFL1-L1*, *GhTFL1-L2*, *GhBFT-L2*, and *GhSFT* were involved predominantly in development, with GO processes including meristem maintenance, and leaf- and reproductive-structure development (Fig. 6). Of particular note, the TEOSINTE BRANCHED1, CYCLOIDEA, and PROLIFERATING CELL NUCLEAR ANTIGEN BINDING FACTOR (TCP) transcription factors were predicted to bind the cotton *SFT* (at –300 nts) and *SP* (at –700 nts) promoters. TCPs govern key plant developmental processes such as regulating branching patterns (Aguilar-Martínez *et al.*, 2007), and differentially interact with AtFT and AtTFL1 (Ho and Weigel, 2014), thereby preventing premature flowering in axillary meristems (Niwa *et al.*, 2013). DNA-binding One zinc Finger (DOF) transcription factors are predicted to bind the cotton *SFT*, *TFL1-L1*, and *TFL1-L2* promoters. DOF transcription factors regulate the formation and function of vascular tissues (Papi *et al.*, 2002), and this is consistent with the vascular staining patterns observed with the *GhSFT*_*pro*_*::uidA*, *GhTFL1-L1*_*pro*_*::uidA*, and *GhTFL1-L2*_*pro*_*::uidA* lines. Transcriptional regulators of *GhBFT-L1*, *GhMFT-L1*, and *GhMFT-L2* were not associated with developmental processes.

## Discussion

Plant architecture is fundamental to crop productivity, and impacts on harvest time, yields, and crop management strategies. The balance between determinate and indeterminate growth is influenced by *CETS* gene activities, and current hypotheses consider that the local balance between FT and TFL1 (and their homologs in other species) is key to achieving this. Consistent with this hypothesis, the domestication of many crops favored architectures amenable to agriculture, such as shorter plants that flowered earlier or independently of day length, and involved artificial selection at *FT*- and *TFL1*-like loci. For example, the transition from indeterminate to the shorter and more lodging-resistant determinate soybean (*Glycine max*) resulted from artificial selection of specific nucleotide substitutions in *GmTFL1* (Tian *et al.*, 2010).

We have previously demonstrated in cotton that members of this gene family coordinate determinate and indeterminate growth patterns, by regulating monopodial and sympodial branching patterns, flowering time, leaf shape, and stem growth (McGarry *et al.*, 2016). *GhSFT* overexpressed from a viral vector in cotton uncoupled flowering from the constraint of photoperiod, and accelerated the transition to flowering in ancestral and domesticated cotton accessions. Fruiting branches terminated with clusters of flowers instead of initiating the next sympodial unit. Silencing *GhSP* by virus-induced gene silencing resulted in an extreme form of determinate growth: the main stem terminated by the 5th node with a terminal flower, and all axillary meristems converted to flowers instead of initiating branches. These results supported a model where the balanced activities of these two gene products are required for indeterminate and determinate growth.

Here, we have expanded our understanding of the *CETS* family in regulating cotton growth by analysing all members. Like closely related jute and cocoa, cotton has a single gene in the *FT*-like clade, *GhSFT*, which is responsible for stimulating the transition to flowering and for regulating sympodial growth in cotton (McGarry *et al.*, 2016). Expressing a genomic clone of *GhSFT* to ~2 kb of upstream and 1 kb of downstream native regulatory sequences rescued the *Atft-10* flowering defect (Fig. 2), and *GhSFT*_*pro*_*::uidA* produced weak GUS activity in the vasculature of transgenic Arabidopsis (Fig. 5). Although extrapolated in a heterologous system, our findings contrast with characterizations of *AtFT* where 5.7 kb of upstream sequence is required to complement the *ft* mutant. Truncating the *AtFT* promoter to 4 kb or less, which excludes *CONSTANS*-responsive elements, does not complement the flowering defect nor confer vascular expression (Adrian *et al.*, 2010). While it is likely that additional *cis*-elements and *trans*-factors regulate *GhSFT* expression in cotton, our findings suggest *GhSFT* and *AtFT* differ in their regulation, and the variation in upstream sequences may contribute to the responses of cotton and Arabidopsis to different photoperiods. Our use of heterologous expression of cotton genes in Arabidopsis warrants discussion (Hernandez-Garcia and Finer, 2014). This approach is advantageous for non-model plants such as cotton as collections of mutants are not readily available, and targeted mutagenesis and regeneration of transformants is not trivial. A critical limitation of the approach is that the presence or absence of different regulators in Arabidopsis and cotton could impact on spatial expression patterns. However, regulatory cascades can be highly conserved and, as an example, the galactinol synthase promoter from melon (*Cucumis melo*) drives minor vein-specific expression in transgenic tobacco (*Nicotiana tabacum*) and Arabidopsis, two species with different phloem-loading biochemistries and vein physiologies (Haritatos *et al.*, 2000; Ayre *et al.*, 2003).

Phylogenetic analyses suggested more extensive duplications occurred within the *TFL1*-like clade in cotton, and that these events were specific to the cotton lineage as cocoa and jute each had a single *TFL1*-like and *BFT*-like gene (Supplementary Fig. S2). Genes of the *Gossypium TFL1*-like clade have different functions in regulating growth. Ectopic expression of all genes of the *TFL1*-like clade delayed developmental transitions in transgenic Arabidopsis (Fig. 1). This is consistent with reports that *AtTFL1* can act outside of its expression domain to repress flowering, as demonstrated when expressed from the *LEAFY*, *APETALA 1*, and *AINTEGUMENTA* promoters (Baumann *et al.*, 2015). Finer resolution of genetic activities was achieved using native regulatory elements to rescue the early-flowering phenotype of the *Attfl1-14* mutant (Fig. 3). Of these, only *GhSP*, *GhTFL1-L2*, and *GhBFT-L2* demonstrated partial rescue, and supporting this, the predicted polypeptides interacted with the cotton FD in yeast two-hybrid assays (Fig. 4). This suggests that differences in regulatory elements led to functional divergence between paralogous genes. This was particularly evident with the *GhBFT* paralogs: *GhBFT-L1*_*pro*_*::GhBFT-L1* did not rescue the early-flowering *Attfl1-14* (Fig. 3); *GhBFT-L1*_*pro*_*::uidA* transgenic plants did not produce GUS staining patterns at any developmental stage tested (Fig. 5); and comparative evolutionary analysis did not identify putative transcription factor binding sites conserved between these promoters (Supplementary Fig. S5). This suggests that, following the gene duplication event, *GhBFT-L1* became uncoupled from its original function and may be undergoing neo-functionalization.

The incomplete rescue of *Attfl1-14* by *GhSP*, *GhTFL1-L2*, and *GhBFT-L2* suggests that additional regulatory sequences probably govern nuanced expression, but that these genes designate meristem identity. Correct spatiotemporal patterning of *AtTFL1* expression is critical for its complex activities in regulating meristem indeterminacy and inflorescence architecture (Baumann *et al.*, 2015; Serrano-Mislata *et al.*, 2016). *AtTFL1* is weakly expressed in the shoot apical meristem after germination but is up-regulated at the floral transition in the switch to inflorescence identity (Bradley *et al.*, 1997). In axillary meristems, *AtTFL1* expression starts strongly and decreases as the axillary shoot elongates (Ratcliffe *et al.*, 1999; Conti and Bradley, 2007). An in-depth analysis of the *cis*-regulatory regions of *AtTFL1* has demonstrated that both the 5′ and 3′ intergenic regions are required to complement the *tfl1-1* mutant phenotype and reproduce the appropriate mRNA expression pattern (Serrano-Mislata *et al.*, 2016). As little as 0.3 kb upstream of the start codon is required for *TFL1* expression, and between 3.3–3.6 kb of sequence downstream of the stop codon are needed for appropriate transcript levels (Serrano-Mislata *et al.*, 2016). That expression of *GhSP*, *GhTFL1-L2*, and *GhBFT-L2* from cotton regulatory sequences restored some level of the *Attfl1-14* developmental defect (Fig. 3) supports the conservation of some regulatory elements among the cotton genes and *AtTFL1*.

The fact that *GhTFL1-L2* and *GhBFT-L2* exhibited such different spatial expression patterns from *GhSP* is particularly intriguing. The *GhTFL1-L2*_*pro*_*::uidA* and *GhBFT-L2*_*pro*_*::uidA* transgenic plants demonstrated an unexpected vascular expression pattern whereas *GhSP*_*pro*_*::uidA* lines showed GUS activity in meristems as predicted (Fig. 5). The vascular patterns suggest these may be anti-florigens; that is, indeterminate factors produced in leaves and transported to meristems where they inhibit determinate growth. As an example, the chrysanthemum (*Chrysanthemum seticuspe*) antiflorigen *CsAFT* is expressed in leaves under non-inductive photoperiods, and antagonizes the florigenic activity of CsFTL3 to inhibit flowering (Higuchi *et al.*, 2013). The expression patterns in heterologous Arabidopsis were consistent with and supported by up-regulation of *GhTFL1-L2* and *GhBFT-L2* expression in the main stem meristems of the photoperiodic cotton TX701 grown under non-inductive long days, whereas *GhSP* was more strongly expressed in those meristems of TX701 grown under short days (Table 1). In addition, the *trans*-regulation analysis (Fig. 6), although not a substitution for *in vitro* or *in vivo* binding assays, predicted the binding of different transcription factors in the upstream sequences of *GhSP*, *GhTFl1-L2*, and *GhBFT-L2*, further supporting distinctions among these genes.

Our characterizations of the cotton *CETS* gene family suggest that they may contribute disparately to plant architecture. Genes of the *TFL1*-like clade are differentially expressed in cotton, influence flowering time distinctly, and demonstrate dissimilarities in their regulation. These results may aid in developing strategies to stably control the ratio of vegetative to reproductive growth for crop management.

## Supplementary data

Supplementary data are available at *JXB* online.

Table S1. Cotton *CETS* genes identified from recent assemblies of *G. arboreum*, *G. raimondii*, and *G. hirsutum*.

Table S2. Oligonucleotides used to amplify the *GhCETS* coding sequences for cloning in pART7 and pGBKT7.

Table S3. Oligonucleotides used to amplify ~2 kb of promoter to drive *uidA* expression in *CETSp::GUS* experiments.

Table S4. Oligonucleotides used for yeast homologous recombination.

Table S5. Oligonucleotides used to amplify up to 200 nts of target sequence by RT-qPCR.

Table S6. Predicted transcription factor binding sites.

Fig. S1. CETS polypeptide alignment.

Fig. S2. Phylogenetic tree showing that duplications in the cotton *CETS* gene family are not observed in closely related malvales.

Fig. S3. Spatial expression profiles of *CETS* genes in cotton.

Fig. S4. Ectopic expression of genes from the *GhTFL1*-like clade produces the I1* phase, with nodes becoming increasingly more flower-like.

Fig. S5. Comparative promoter analysis of the *GhTFL1* and *GhBFT* paralogs.

Supplementary Tables and Figures
