# Supplementary material for: Cotton CENTRORADIALIS/TERMINAL FLOWER 1/SELF-PRUNING genes functionally diverged to differentially impact plant architecture
Source: J Exp Bot. 2018 Sep 10;69(22):5403–17. doi: 10.1093/jxb/ery324 (PMC6255698; doi:10.1093/jxb/ery324)
Supplement: Supplementary Tables and Figures [file ery324_suppl_supplementary_tables_figures.pdf]

Supplementary Information

Supplementary Table S1: Cotton *CETS* genes identified from recent assemblies of *G. arboreum, G. raimondii,* and *G. hirsutum*. Shown are the gene identifiers from recent cotton genome sequences. The *GhTFL1-L2* homeolog from the A_t subgenome was not determined from the most recent *G. hirsutum* assembly (Tx-JGI).

Supplementary Table S2: Oligonucleotides used to amplify the *GhCETS* coding sequences for cloning in pART7 and pGBKT7. Primers cannot distinguish between homeologs. Cloning in pART7 used primers listed as gene name EcoRI ATG fwd and gene name XbaI STOP rev. Cloning in pGBKT7 used primers indicated as gene name EcoRI ATG fwd with gene name SalI STOP rev or gene name PstI STOP rev, and TFL1-L2 NdeI fwd with TFL1-L2 BamHI rev.

Supplementary Table S3: Oligonucleotides used to amplify ~ 2 kb of promoter to drive *uidA* expression in *CETSp:GUS* experiments.

Supplementary Table S4: Oligonucleotides used for yeast homologous recombination.

Supplementary Table S5: Oligonucleotides used to amplify up to 200 nts of target sequence by RT-qPCR. Primers cannot distinguish between homeologs.

Supplementary Table S6: Predicted transcription factor binding sites. *CETS* promoters from *G. raimondii*, *G. arboreum*, and *G. hirsutum* genomes were used as input to predict conserved transcription factors using a *G. raimondii* transcription factor database. Descriptions of transcription factors were mined from Phytozome (https://phytozome.jgi.doe.gov/pz/portal.html). The closest Arabidopsis homologs were identified from BLAST queries; homologs in red font indicate orthologous genes based upon reciprocal BLASTp searches. Binding sites reflect distance in nucleotides from the 5' promoter; strand binding is shown as "+" or "-".

Supplementary Figure S1: CETS polypeptide alignment. *G raimondii* (Gr), *G. arboreum* (Ga), and *G. hirsutum* (Gh, NAU-NBI) CETS protein sequences are aligned with CETS from Arabidopsis (*Arabidopsis thaliana*, At), jute (*Corchorus olitorius* and *Corchorus capsularis,* Co and Cc), cacao (*Theobroma cacao,* Tc), tomato (*Solanum lycopersicum* and *Solanum pimpinellifolium*, Sl and Sp), and moss (*Physcomitrella patens*, Pp) using Clustal Omega. "*****" indicates identical residues, "**:**" indicates conserved residues (scoring > 0.5 in the Gonnet PAM

250 matrix), and "**.**" denotes conservation between amino acids with weakly similar properties (scoring < 0.5 in the Gonnet PAM 250 matrix).

Supplementary Figure S2: Duplications in the cotton *CETS* gene family are not observed in closely related malvales. Shown is a phylogenetic tree constructed from the predicted polypeptide sequences of the *G. raimondii* (Gr), *G. arboreum* (Ga), and *G. hirsutum* (Gh, NAU-NBI assembly) homologs with Arabidopsis (*Arabidopsis thaliana,* At*)*, tomato (*Solanum lycopersicum*, Sl), cacao (*Theobroma cacao*, Tc), jute (*Corchorus capsularis* and *Corchorus olitorius*, Cc and Co), and moss (*Physcomitrella patens*, Pp) CETS. Red arrows point to gene duplications in *Gossypium*. The scale bar represents amino acid substitution frequency determined by the Poisson correction method. Evolutionary analyses were conducted in MEGA7. Tree branches are labeled with percentages of 1000 iterations support, and the tree is drawn to scale with branch lengths measured in the number of substitutions per site.

Supplementary Figure S3: Spatial expression profiles of *CETS* genes in cotton. The relative expression of *GhBFT-L1*, *GhTFL1-L1*, *GhTFL1-L2*, *GhMFT-L1*, and *GhMFT-L2* in the shoots of day-neutral DP61 and photoperiodic TX701 plants grown under inductive short-days (SD) or non-inductive long days (LD) was quantified by RT-qPCR. Expression was determined in source leaves, newly emerged sink leaves, immature floral buds ("square"), the monopodial main stem apex, the vegetative branch apex, and a reproductive fruiting branch apex, and compared with expression of *GhUBQ10* in these tissues.

Supplementary Figure S4: Ectopic expression of genes from the *GhTFL1*-like clade produces the I1* phase, with nodes becoming increasingly flower-like. Shown in this $35S_{pro}$*:GhBFT-L1* plant, the basal nodes appear branch-like, and bear a few leaves before terminating with a floral cluster. Progressing distally along the inflorescence: nodes no longer have leaves; floral clusters arise from sepal-like structures; floral clusters arise from unfused carpels; and finally at the plant apex, normal flowers form.

Supplementary Figure S5: Comparative promoter analysis of the *GhTFL1* and *GhBFT* paralogs. Shown are R-vista alignments of the 2 kb promoters from **(A)** *GhTFL1-L1* (Gohir.D09G135900) and *GhTFL1-L2* (Gohir.D04G100700), and **(B)** *GhBFT-L1* (Gohir.D08G112000) and *GhBFT-L2* (Gohir.D11G009200). Evolutionarily conserved non-coding sequences shared between the paralogs are shown as red peaks.

Supplementary Table S1: Cotton *CETS* genes identified from recent assemblies of *G. arboreum, G. raimondii,* and *G. hirsutum*.

Assigned gene name	*G. arboreum* A2 BGI-CGP assembly v2.0 (annot v1.0)	*G raimondii* D5 JGI assembly v2.0 (annot v2.1)	*G. hirsutum* AD1 NAU-NBI assembly v1.1 (annot v1.1)		*G. hirsutum* AD1 genome Tx-JGI assembly v1.0 (annot v1.1)	
			D_t subgenome	A_t subgenome	D_t subgenome	A_t subgenome
MFT-L1	Cotton_A_13046	Gorai.006G192300.1	Gh_D09G1658	Gh_A09G2391	Gohir.D09G170700	Gohir.A09G175400
MFT-L2	Cotton_A_04728	Gorai.009G174600.1	Gh_D05G1586	Gh_Sca004734G03	Gohir.D05G169400	Gohir.A05G166400
SFT	Cotton_A_05804	Gorai.004G264600.1	Gh_D08G2407	Gh_A08G2015	Gohir.D08G248000	Gohir.A08G227700
TFL1-L1	Cotton_A_13428	Gorai.006G155800.1	Gh_D09G1320	Gh_A09G2442	Gohir.D09G135900	Gohir.A09G138800
TFL1-L2	Cotton_A_31651	Gorai.009G403800.1	Gh_D04G0971	Gh_A04G0520	Gohir.D04G100700	
BFT-L2	Cotton_A_07540	Gorai.007G010800.1	Gh_D11G0092	Gh_A11G0088	Gohir.D11G009200	Gohir.A11G009900
BFT-L1	Cotton_A_39415	Gorai.004G120400.1	Gh_D08G1087	Gh_Sca15601	Gohir.D08G112000	Gohir.A08G100900
SP	Cotton_A_09584	Gorai.001G121800.1	Gh_D07G1075	Gh_A07G0997	Gohir.D07G113500	Gohir.A07G109700

Shown are the gene identifiers from recent cotton genome sequences. The *GhTFL1-L2* homeolog from the A_t subgenome was not determined from the most recent *G. hirsutum* assembly (Tx-JGI).

Supplementary Table S2: Oligonucleotides used to amplify the *GhCETS* coding sequences for cloning in pART7 and pGBKT7.

OLIGONUCLEOTIDE	SEQUENCE (5')
MFT-L1 EcoRI ATG fwd	ctcgtgGAATTCatggctgcctccgttgatcctc
MFT-L1 XbaI STOP rev	ctcgtgTCTAGAtcaacgccttcggctgacg
MFT-L1 Pst1 STOP rev	ctcgtgCTGCAGtcaacgccttcggctgacg
MFT-L2 EcoRI ATG fwd	ctcgtgGAATTCatggcccggtccgttgaacca
MFT-L2 XbaI STOP rev	ctcgtgTCTAGActaacgtttcttagctgctgg
MFT-L2 Sal1 STOP rev	ctcgtgGTCGACctaacgtttcttagctgctgg
SFT EcoRI ATG fwd	ctcgtgGAATTCatgcctagagatagagatcctttg
SFT XbaI STOP rev	ctcgtgTCTAGAtcatgtcctacggccacc
SFT Sal1 STOP rev	ctcgtgGTCGACtcatgtcctacggccacc
TFL1-L1 EcoRI ATG fwd	ctcgtgGAATTCatggcaagggaagtagagcctc
TFL1-L1 XbaI STOP rev	ctcgtgTCTAGAtcaacgtcttcttgcagctg
TFL1-L1 Pst1 STOP rev	ctcgtgCTGCAGtcaacgtcttcttgcagctg
TFL1-L2 EcoRI ATG fwd	ctcgtgGAATTCatgggagagcctctcattgttg
TFL1-L2 XbaI STOP rev	ctcgtgTCTAGAttagcgtctccttgcagcag
TFL1-L2 NdeI fwd	ctcgtgCATATGggagagcctctcattgttgg
TFL1-L2 BamHI rev	ctcgtgGGATCCttagcgtctccttgcagcag
BFT-L1 EcoRI ATG fwd	ctcgtgGAATTCatgtcaagagtccccgaccca
BFT-L1 XbaI STOP rev	ctcgtgTCTAGAtcatcttcttgatcttgcgg
BFT-L1 SalI stop rev	ctcgtgGTCGACtcatcttcttgatcttgcgg
BFT-L2 EcoRI ATG fwd	ctcgtgGAATTCatgtcaagggtccctgagccac
BFT-L2 XbaI STOP rev	ctcgtgTCTAGAttatcttcttcttgcagcagtt
BFT-L2 Sal1 STOP rev	ctcgtgGTCGACttatcttcttcttgcagcagtt
SP EcoRI ATG fwd	ctcgtgGAATTCatggcaaaactgtcagatcctctt
SP XbaI STOP rev	ctcgtgTCTAGAttagcgtcttctagcagctg
SP SalI STOP rev	ctcgtgGTCGACttagcgtcttctagcagctg

Primers cannot distinguish between homeologs. Cloning in pART7 used primers listed as gene name EcoRI ATG fwd and gene name XbaI STOP rev. Cloning in pGBKT7 used primers indicated as gene name EcoRI ATG fwd with gene name SalI STOP rev or gene name PstI STOP rev, and TFL1-L2 NdeI fwd with TFL1-L2 BamHI rev.

Supplementary Table S3: Oligonucleotides used to amplify ~ 2 kb of promoter to drive *uidA* expression in *CETSp:GUS* experiments.

OLIGONUCLEOTIDE	SEQUENCE (5')
GhMFT-L1p SbfI nt -1923 fwd	atatctCCTGCAGGtttagatctctaactgagttggtgagatg
GhMFT-L1p XmaI nt -1 rev	tCCCGGGtctagagggagaaagaggagtgggggtggggtgca
GhMFT-L2p SbfI nt -2231 fwd	ctcagaCCTGCAGGcctttgaagccctcttcctttt
GhMFT-L2p XmaI nt -1 rev	ggaccgCCCGGGagtgtgttggactagacctg
GhSFTp SbfI nt -1806 fwd	ctcgtgCCTGCAGGcctaagcctaaaaatcagctaccctac
GhSFTp XmaI nt -1 rev	atctctCCCGGGgatatcgctatttggtcttactgtg
GhTFL1-L1p SbfI nt -2076 fwd	aagcttCCTGCAGGgagacttgtataggttttgc
GhTFL1-L1p XmaI nt -1 rev	ttccctCCCGGGttgaggagttctgaatgaaag
GhTFL1-L2p SbfI nt -2124 fwd	ctcgtgCCTGCAGGtgcaaaattttggaggctacaact
GhTFL1-L2p XmaI nt -1 rev	ctcgtgCCCGGGtggtgacactgaatgaaaagaagaga
GrBFT-L1p fwd nt -2013	ggtgttcttatgtagtgacaccgattatttaaaCCTGCAGGatgtcaatttgacgatcaatg tcg
GhBFT-L1p XmaI nt -1 rev	ctcgtgCCCGGGgatatatatatttttagctaatg
GhBFT-L2p SbfI nt -1959 fwd	aagcttCCTGCAGGggatccaaagagtgatttaacc
GhBFT-L2p XmaI nt -1 rev	gaccctCCCGGGgatgaacaagacgatatgtatg
GhSPp SbfI nt -2031 fwd	aagcttCCTGCAGGgggtatggcatgagaaatcacc
GhSPp XmaI nt -1 rev	cagtttCCCGGGcccacaaactaatataacactgg

Supplementary Table S4: Oligonucleotides used for yeast homologous recombination.

OLIGONUCLEOTIDE	SEQUENCE (5’)
GrMFT-L1p hr fwd	ggtgttcttatgtagtgacaccgattatttaaacctgcaggatggatttgtattacctctatc
GrMFT-L1p hr rev	ggatcaacggaggcagccatgggagaaagaggagtgggggtgcagt
GrMFT-L1 hr fwd	actgcacccacccccactcctctttctcccatggctgcctccgttgatcc
GrMFT-L1 hr rev	tcacccgggacgccttcggctgacgggctc
GrMFT-L1t hr fwd	gagctcatctcccacacacacttctctc
GrMFT-L1t hr rev	gtggtggtggctagcgttaacactagtcagatctaccatgggaattccatctaccttttaattggaag
bGrMFT-L1 fwd	ctgtctatttcaacgcccaaaaagagcccgtcagccgaaggcgtcccgggtgagcggccgcgag
bGrMFT-L1 rev	ccagttttatatttatatattttatatatagagagagagagagaagtgtgtgtgggagatgagctcgcggccgctcac
bGrMFT-L1 rev	ccagttttatatttatatattttatatatagagagagagagagaagtgtgtgtgggagatgagctcgcggccgctcac
GrMFT-L2p hr fwd	ggtgttcttatgtagtgacaccgattatttaaacctgcaggatggcttgatttaaaatctccg
GrMFT-L2p hr rev	gtggttcaacggaccgggccatagtgtgttggactagacctgcg
GrMFT-L2 hr fwd	cgcaggtctagtccaacacactatggcccggtccgttgaaccac
GrMFT-L2 hr rev	ctacccgggacgtttcttagctgctggctcc
GrMFT-L2t hr fwd	gagctcctcatagcttacagtgcattatttgg
GrMFT-L2t hr rev	gtggtggtggctagcgttaacactagtcagatctaccatgggaattcccaatgtataggagtgaagc
bGrMFT-L2 fwd	gcagtgtatttcaattctcaaaaggagccagcagctaagaaacgtcccgggtaggcggccgcgag
bGrMFT-L2 rev	gtattacagaacctaccaaataatgcactgtaagctatgaggagctcgcggccgcctac
GrSFTp fwd	ggtgttcttatgtagtgacaccgattatttaaacctgcaggatgcctaaaaatcagctaccctacg
GrSFTp rev	ggatctctatctctaggcatgatatcgctatttggtcttac
GrSFT hr fwd	gtaagaccaaatagcgatatcatgcctagagatagagatcc
GrSFT hr rev	tgtcctacggccaccggatccac
GrSFTt hr fwd	gagctcaataaataatattgttgttgttgatc
GrSFTt hr rev	gtggtggtggctagcgttaacactagtcagatctaccatgggaattccacatttattcaatttggtctc
bGrSFT fwd	ctgccagagggagagtggatccggtggccgtaggacacccgggtaggcggccgcgag
bGrSFT rev	cttgaaatcaaacatgatcaacaacaacaatattatttattgagctcgcggccgcctac
GrTFL1-L1p fwd	ggtgttcttatgtagtgacaccgattatttaaacctgcaggatgcattgattgaacttacttgctc
GrTFL1-L1p rev	ggctctacttcccttgccatttgaggagttctgaatgaaagaaagag
GrTFL1-L1 hr fwd	ctctttctttcattcagaactcctcaaatggcaagggaagtagagcc
GrTFL1-L1 hr rev	tcacccgggacgtcttcttgcagctgtttctc
GrTFL1-L1t hr fwd	gagctcttaacctgcacaaaagtatatctg
GrTFL1-L1t hr rev	gtggtggtggctagcgttaacactagtcagatctaccatgggaattcaggtgagtttgtgcctgattt
bGrTFL1-L1 fwd	ctgtgtatttcaatgctcaaagagaaacagctgcaagaagacgtcccgggtgagcggccgcgag

bGrTFL1-L1 rev	caagaacaccccatcacagatatacttttgtgcaggttaagagctcgcggccgctcac
GrTFL1-L2p hr fwd	ggtgttcttatgtagtgacaccgattatttaaacctgcaggatgattacttagtatattattcag
GrTFL1-L2p hr rev	actcccccaacaatgagagg
GrTFL1-L2 hr fwd	ttcattcagtgtcaccaagaatgggagagcctctcattgttgg
GrTFL1-L2 hr rev	ttacccggggcgtctccttgcagcag
GrTFL1-L2t hr fwd	gagctcttaaaccttcaaaactcaagaaag
GrTFL1-L2t hr rev	gtggtggtggctagcgttaacactagtcagatctaccatgggaattcgagaggttccttaaacgtt cag
bGrTFL1-L2 fwd	gctgtttatttcaatgcacgaagagaaactgctgcaaggagacgccccgggtaagcggccgcg ag
bGrTFL1-L2 rev	ccaatccaaatggaacgttctttcttgagttttgaaggtttaagagctcgcggccgcttac
GrBFT-L1p hr fwd	ggtgttcttatgtagtgacaccgattatttaaacctgcaggatgtcaatttgacgatcaatgtcg
GrBFT-L1p hr rev	gggtcggggactcttgacatgatatatatatttttagctaatgaatattgc
GrBFT-L1 hr fwd	gcaatattcattagctaaaaatatatatatcatgtcaagagtccccgaccc
GrBFT-L1 hr rev	tcacccgggtcttcttgatcttgcggcag
GrBFT-L1t hr fwd	gagctcctctgccacttccataataatatatac
GrBFT-L1t hr rev	gtggtggtggctagcgttaacactagtcagatctaccatgggaattcccctcaagatagctagatt aagc
bBFT-L1 fwd	gcagtttacttcaatgcccagagagaaactgccgcaagatcaagaagacccgggtgagcggcc gcgag
bBFT-L1 rev	cagtagtaaattatgtatgtatatattattatggaagtggcagaggagctcgcggccgctcag
GrBFT-L2p hr fwd	ggtgttcttatgtagtgacaccgattatttaaacctgcaggatgagaatagttaccaaattaaggatc caaagagtg
GrBFT-L2p hr rev	ggctcagggacccttgacatgatgaacaagacgatatgtatg
GrBFT-L2 hr fwd	catacatatcgtcttgttcatcatgtcaagggtccctgagcc
GrBFT-L2 hr rev	ttacccgggtcttcttcttgcagcagtttctc
GrBFT-L2t hr fwd	gagctcctatggctgccccatagacattaag
GrBFT-L2t hr rev	gtggtggtggctagcgttaacactagtcagatctaccatgggaattcggcatgcttaattgggtag g
bGrBFT-L2 fwd	cttcaatgcccagagagaaactgctgcaagaagaagacccgggtaagcggccgcgag
bGrBFT-L2 rev	ccttcattttattatacttaatgtctatggggcagccataggagctcgcggccgcttac
GrSPp hr fwd	ggtgttcttatgtagtgacaccgattatttaaacctgcaggatgggtatggcatgagaaatcaccat gtatc
GrSPp hr rev	ggatctgacagttttgccatcccacaaactaatataacactgg
GrSP hr fwd	ccagtgttatattagtttgtgggatggcaaaactgtcagatcc
GrSP hr rev	ttacccggggcgtcttctagcagctgtttccc
GrSPt hr fwd	gagctcaacataaagtggttcaccaatggatc
GrSPt hr rev	gtggtggtggctagcgttaacactagtcagatctaccatgggaattcttcaactgggtttttgttcttc
bGrSP fwd	ctgtctatttcaatgctcaaagggaaacagctgctagaagacgccccgggtaagcggccgcga g
bGrSP rev	gggcaaacatcattgatccattggtgaaccactttatgttgagctcgcggccgcttac

Supplementary Table S5: Oligonucleotides used to amplify up to 200 nts of target sequence by RT-qPCR.

OLIGONUCLEOTIDE	SEQUENCE (5')
UBQ10 fwd	ggtttccgaatggtcttttg
UBQ10 rev	tttagccactgcatcacagc
GhTFL1-L1 fwd nt 234	cctggacctagtgatccttact
GhTFL1-L1 rev nt 336	catagctcaccacttcccttc
GhTFL1-L2 nt 263 fwd	acatcccaggcacaacagat
GhTFL1-L2 nt 384 rev	acctgtcgacgtttctgctt
GhBFT-L1 fwd nt 322	cttcctttgggagggaagtt
GhBFT-L1 rev nt 433	ggtggcctcactgtttgtct
GhBFT-L2 fwd nt 321	gtttcgtttgggagggaagt
GhBFT-L2 rev nt 448	taatccctcgaagacggtga
GhMFT-L1 fwd nt 268	gacattcctggaggaaccaa
GhMFT-L1 rev nt 386	ccctttgttggaacagcac
GhMFT-L2 fwd nt 278	ttccagagggacaagatgct
GhMFT-L2 rev nt 394	ccattgctccttcctgctta

Primers cannot distinguish between homeologs.

Supplementary Table S6: Predicted transcription factor binding sites.

GhCETS Promoter	Conserved TF Identifier	Conserved TF Description (Arabidopsis homolog)	TF Family	Bound Promoter	Binding Site
SFT	(a) Gorai.001G199200	WUSCHEL-related homeobox 10-related (AtWOX13)	WOX	GaSFT<br>GhSFT-A<br>GhSFT-D<br>GrSFT	1053 - 1062 +<br>1054 - 1063 +<br>1039 - 1048 +<br>1036 - 1045 +
	(a) Gorai.006G188900<br>(b) Gorai.009G162500<br>(c) Gorai.009G319300<br>(d) Gorai.011G040200<br>(e) Gorai.011G067500	(AtDof5.6)<br>(AtCOGWHEEL1)<br>Dof2.4 (AtDof2.4)<br>(AtCOGWHEEL1)<br>(AtDof2)	Dof	GaSFT<br>GhSFT-A<br>GhSFT-D<br>GrSFT	1290 - 1310 +<br>1291 - 1311 +<br>1289 - 1309 +<br>1288 - 1308 +
	(a) Gorai.005G087600	PISTILLATA (AtPI)	MIKC-MADS	GaSFT<br>GhSFT-A<br>GhSFT-D<br>GrSFT	1352 - 1365 +<br>1353 - 1366 +<br>1351 - 1364 +<br>1350 - 1363 +
	(a) Gorai.005G087600	PISTILLATA (AtPI)	MIKC-MADS	GaSFT<br>GhSFT-A<br>GhSFT-D<br>GrSFT	1377 - 1390 +<br>1378 - 1391 +<br>1376 - 1389 +<br>1375 - 1388 +
	(a) Gorai.009G333800	GT-1 related (AtGT-1)	trihelix	GaSFT<br>GhSFT-A<br>GhSFT-D<br>GrSFT	1392 - 1399 +/-<br>1393 - 1400 +/-<br>1391 - 1398 +/-<br>1390 - 1397 +/-
	(a) Gorai.008G115200	(AtSOC1)	MIKC-MADS	GaSFT<br>GhSFT-A	1616 - 1636 -<br>1617 - 1637 -

				GhSFT-D<br>GrSFT	1617 - 1637 -<br>1619 - 1639 -
	(a) Gorai.011G067500<br>(b) Gorai.011G040200<br>(c) Gorai.009G319300<br>(d) Gorai.009G162500<br>(e) Gorai.006G188900<br>(f) Gorai.001G067000<br>(g) Gorai.003G021000<br>(h) Gorai.006G173700<br>(i) Gorai.008G193700<br>(j) Gorai.009G100100	(AtDof2)<br>(AtCOGWHEEL1)<br>Dof2.4 (AtDof2.4)<br>(AtCOGWHEEL1)<br>(AtDof5.6)<br>(AtOBF BINDING PROTEIN 4)<br>Dof 3.4<br>(AtOBF BINDING PROTEIN 1)<br>(AtDof2.4)<br>CELLGROWTH DEFECT FACTOR2 related (AtCDF3)<br>(AtOBF BINDING PROTEIN 3)	Dof	GaSFT<br>GhSFT-A<br>GaSFT<br>GhSFT-A	1615(25) - 1635 (46) +<br>1615(25) - 1635 (46) +<br>1615(25) - 1635 (46) +<br>1615(25) - 1635 (46) +
	(a) Gorai.001G072200	(AtTCP14)	tcp	GaSFT<br>GhSFT-A<br>GhSFT-D<br>GrSFT	1713 - 1732 -<br>1714 - 1733 -<br>1714 - 1733 -<br>1716 - 1735 -
	(a) Gorai.008G086400	REGULATOR OF AXILLARY MERISTEMS2 (AtRAX3)	MYB	GaSFT<br>GhSFT-A<br>GhSFT-D<br>GrSFT	1720 - 1732 -<br>1721 - 1733 -<br>1721 - 1733 -<br>1723 - 1735 -
SP	(a) Gorai.002G124900	BPC6-related (AtBPC6)	BBR-BPC	GaSP<br>GhSP-A<br>GhSP-D<br>GrSP	438 - 458 -<br>373 - 393 -<br>334 - 354 -<br>258 - 278 -
	(a) Gorai.002G171200	BPC1-related (AtBPC2)	BBR-BPC	GaSP<br>GhSP-A	436 -459 +<br>371 - 394 +

				GhSP-D<br>GrSP	332 - 355 +<br>258 - 281 +
	(a) Gorai.006G259700	ARABIDOPSIS RESPONSE REGULATOR1-related (AtARR1)	ARR-B	GaSP<br>GhSP-A<br>GhSP-D<br>GrSP	453 - 462 -<br>388 - 397 -<br>349 - 358 -<br>338 -347 -
	(a) Gorai.011G086900	TCP20-related (AtTCP20)	TCP	GaSP<br>GhSP-A<br>GhSP-D<br>GrSP	1294 - 1314 +<br>1294 - 1314 +<br>1290 - 1310 +<br>1277 - 1297 +
	(a) Gorai.006G197000<br>(b) Gorai.009G153900<br>(c) Gorai.005G211900<br>(d) Gorai.006G043800<br>(e) Gorai.013G068600	TCP19 (AtTCP19)<br>TCP2-related (AtTCP2)<br>TCP21-related (AtTCP7)<br>TCP20-related (AtTCP20)<br>TCP21-related (AtTCP7)	TCP	GaSP<br>GhSP-A<br>GhSP-D<br>GrSP	1304 - 1311 -<br>1304 - 1311 -<br>1300 - 1307 -<br>1287 - 1294 -
	(a) Gorai.001G200400	TCP1 (AtTCP1)	TCP	GaSP<br>GhSP-A<br>GhSP-D<br>GrSP	1303 - 1332 +<br>1303 - 1332 +<br>1299 - 1328 +<br>1286 - 1315 +
	(a) Gorai.003G021000<br>(b) Gorai.009G100100	Dof 3.4 (AtOBF BINDING PROTEIN 1)<br>(AtOBF BINDING PROTEIN 3)	Dof	GaSP<br>GhSP-A<br>GhSP-D<br>GrSP	1941 - 1961 -<br>1941 - 1961 -<br>1940 - 1960 -<br>1941 - 1961 -
	(a) Gorai.011G067500	(AtDof2)	Dof	GaSP<br>GhSP-A<br>GhSP-D<br>GrSP	1950 - 1970 +<br>1950 - 1970 +<br>1949 - 1969 +<br>1950 - 1970 +
	(a) Gorai.008G115200<br>(b) Gorai.N017200	(AtSOC1)<br>AGAMOUS-like AGL11 (AtAG)	MIKC_MADS	GaSP	1941(44) -<br>1961(62) -

				GhSP-A	1941(44) -<br>1961(62) -
				GhSP-D	1940(43) -<br>1960(61) -
				GrSP	1941(47) -<br>1961(62) -
	(a) Gorai.005G087600	PISTILLATA (AtPI)	MIKC_MADS	GaSP	1947 - 1960 +
				GhSP-A	1947 - 1960 +
				GhSP-D	1946 - 1959 +
				GrSP	1947 - 1960 +
TFL1-L1	(a) Gorai.009G319300	Dof2.4 (AtDof2.4)	Dof	GaTFL1-L1	471 - 491 +
				GhTFL-L1-A	476 - 496 +
				GhTFL1-L1-D	480 - 500 +
				GrTFL1-L1	475 - 495 +
	(a) Gorai.002G177000	DELLA protein (AtREPPRESSOR OF GA)	GRAS	GaTFL1-L1	1011 -1030 +
				GhTFL-L1-A	1016 - 1035 +
				GhTFL1-L1-D	1012 - 1031 +
				GrTFL1-L1	1007 - 1026 +
	(a) Gorai.008G115200	(AtSOC1)	MIKC-MADS	GaTFL1-L1	1012 - 1032 -
				GhTFL-L1-A	1017 - 1037 -
				GhTFL1-L1-D	1013 - 1033 -
				GrTFL1-L1	1008 - 1028 -
	(a) Gorai.009G319300	Dof2.4 (AtDof2.4)	Dof	GaTFL1-L1	1480 - 1500 -
				GhTFL-L1-A	1480 - 1500 -
				GhTFL1-L1-D	1485 - 1505 -
				GrTFL1-L1	1480 - 1500 -
	(a) Gorai.011G067500	(AtDof2)	Dof	GaTFL1-L1	1480 - 1500 +
				GhTFL-L1-A	1480 -1500 +
				GhTFL1-L1-D	1484 - 1504 +
				GrTFL1-L1	1479 - 1499 +

	(a) Gorai.005G211900<br>(b) Gorai.008G181600<br>(c) Gorai.013G068600	TCP21-related (AtTCP7)<br>TCP15 (AtTCP15)<br>TCP21-related (AtTCP7)	TCP	GaTFL1-L1<br>GhTFL-L1-A<br>GhTFL1-L1-D<br>GrTFL1-L1	1596 - 1606 -<br>1596 - 1606 -<br>1595 - 1605 -<br>1590 - 1600 -
	(a) Gorai.007G094200<br>(b) Gorai.008G157300<br>(c) Gorai.012G084600	TCP9 (AtTCP9)<br>TCP20-related (AtTCP20)<br>TCP20-related (AtTCP20)	TCP	GaTFL1-L1<br>GhTFL-L1-A<br>GhTFL1-L1-D<br>GrTFL1-L1	1597 - 1606 +<br>1597 - 1606 +<br>1596 - 1605 +<br>1591 - 1600 +
	(a) Gorai.004G263900	BABYBOOM (AtBBM)	AP2	GaTFL1-L1<br>GhTFL-L1-A<br>GhTFL1-L1-D<br>GrTFL1-L1	1781 - 1800 +<br>1781 - 1800 +<br>1780 - 1799 +<br>1755 - 1974 +
	(a) Gorai.002G177000	DELLA protein (AtRGA)	GRAS	GaTFL1-L1<br>GhTFL-L1-A<br>GhTFL-L1-D<br>GhTFL1-L1-D	1789 - 1808 +<br>1789 - 1808 +<br>1791 - 1810 +<br>1786- 1805 +
	(a) Gorai.002G124900	BPC6 (AtBPC6)	BBR-BPC	GaTFL1-L1<br>GhTFL-L1-A<br>GhTFL1-L1-D<br>GrTFL1-L1	1789 - 1809 -<br>1791 - 1811 -<br>1786 - 1806 -<br>1781 - 1801 -
	(a) Gorai.002G124900	BPC6 (AtBPC6)	BBR-BPC	GaTFL1-L1<br>GhTFL-L1-Ap<br>GhTFL1-L1-D<br>GrTFL1-L1	1791 - 1811 -<br>1789 - 1809 -<br>1788 - 1808 -<br>1783 - 1803 -
	(a) Gorai.002G124900	BPC6 (AtBPC6)	BBR-BPC	GaTFL1-L1<br>GhTFL-L1-A<br>GhTFL1-L1-D<br>GrTFL1-L1	1793 - 1813 -<br>1793 - 1813 -<br>1790 - 1810 -<br>1785 - 1805 -
	(a) Gorai.002G171200	BPC1-related (AtBPC2)	BBR-BPC	GaTFL1-L1<br>GhTFL-L1-A	1787 - 1810 +<br>1787 - 1810 +

				GhTFL1-L1-D	1786 - 1809 +
				GrTFL1-L1	1781 - 1804 +
	(a) Gorai.002G171200	BPC1-related (AtBPC2)	BBR-BPC	GaTFL1-L1	1789 - 1812 +
				GhTFL-L1-A	1789 - 1812 +
				GhTFL1-L1-D	1788 - 1811 +
				GrTFL1-L1	1785 - 1808 +
	(a) Gorai.002G171200	BPC1-related (AtBPC2)	BBR-BPC	GaTFL1-L1	1791 - 1814 +
				GhTFL-L1-A	1791 - 18114 +
				GhTFL1-L1-D	1790 - 1813 +
				GrTFL1-L1	1783 - 1806 +
TFL1-L2	(a) Gorai.011G098600	WUSCHEL (AtWUS)	WOX	GaTFL-L2	1094 - 1104 -
				GhTFL1-L2-A	1151 - 1125 -
				GhTFL1-L2-D	1079 - 1089 -
				GrTFL1-L2	1112 - 1122 -
	(a) Gorai.002G115800	Nin-Like PROTEIN4-related (AtNLP4)	Nin-like	GaTFL-L2	1083 - 1097 +
				GhTFL1-L2-A	1104 - 1118 +
				GhTFL1-L2-D	1068 - 1082 +
				GrTFL1-L2	1101 - 1115 +
	(a) Gorai.009G309000	NAC protein 53-related (AtNAC2)	NAC	GaTFL-L2	1069 - 1080 -
				GhTFL1-L2-A	1090 - 1101 -
				GhTFL1-L2-D	1054 - 1065 -
				GrTFL1-L2	1087 - 1098 -
	(a) Gorai.002G234700<br>(b) Gorai.006G188900	Dof1.1-related<br>(AtDof5.6)	Dof	GaTFL-L2	1265(67) -<br>1277(85) -
				GhTFL1-L2-A	1286(88) -<br>1298(1306) -
				GhTFL1-L2-D	1250(52) -<br>1262(70) -
				GrTFL1-L2	1285 - 1295(1305)<br>-

	(a) Gorai.009G319300	Dof2.4 (AtDof2.4)	Dof	GaTFL-L2	1267 - 1287 +
				GhTFL1-L2-A	1288 - 1308 +
				GhTFL1-L2-D	1252 - 1272 +
				GrTFL1-L2	1285 - 1305 +
	(a) Gorai.007G113900	CIRCADIAN CLOCK ASSOCIATED1-related (AtLATE ELOGATED HYPOCOTYL1)	MYB-related	GaTFL-L2	1293 - 1301 -
				GhTFL1-L2-A	1314 - 1322 -
				GhTFL1-L2-D	1278 - 1286 -
				GrTFL1-L2	1311 - 1319 -
	(a) Gorai.004G263900	BBM (AtBBM)	AP2	GaTFL-L2	1411 - 1430 +
				GhTFL1-L2-A	1436 - 1455 +
				GhTFL1-L2-D	1397 - 1416 +
				GrTFL1-L2	1430 - 1449 +
	(a) Gorai.004G263900	BBM (AtBBM)	AP2	GaTFL-L2	1414 - 1433 +
				GhTFL1-L2-A	1438 - 1457 +
				GhTFL1-L2-D	1399 - 1418 +
				GrTFL1-L2	1432 - 1451 +
	(a) Gorai.008G115200	(AtSOC1)	MIKC-MADS	GaTFL-L2	1414 - 1434 -
				GhTFL1-L2-A	1434 - 1454 -
				GhTFL1-L2-D	1398 - 1418 -
				GrTFL1-L2	1431 - 1451 -
	(a) Gorai.001G200400	TCP1 (AtTCP1)	TCP	GaTFL-L2	1676 - 1705 -
				GhTFL1-L2-A	1692 - 1721 -
				GhTFL1-L2-D	1700 - 1729 -
				GrTFL1-L2	1698 - 1727 -
	(a) Gorai.009G289000	TCP16-related (AtTCP23)	TCP	GaTFL-L2	1696 - 1705 +
				GhTFL1-L2-A	1712 - 1721 +
				GhTFL1-L2-D	1720 - 1729 +
				GrTFL1-L2	1718 - 1727 +
BFT-L1	(a) Gorai.008G155600	Ethylene Response Factor(ERF)087	ERF	GaBFT-L1	774 - 788 +

				GhBFT-L1-A<br>GhBFT-L1-D<br>GrBFT-L1	322 - 336 +<br>938 - 952 +<br>872 - 886 +
	(a) Gorai.011G029700	(AtERF12)	ERF/AP2	GaBFT-L1<br>GhBFT-L1-A<br>GhBFT-L1-D<br>GrBFT-L1	774 - 784 -<br>325 - 332 -<br>945 - 952 -<br>879 - 886 -
	(a) Gorai.013G244500	SHINE (AtRelated to AP2.11)	ERF	GaBFT-L1<br>GhBFT-L1-A<br>GhBFT-L1-D<br>GrBFT-L1	774 - 788 -<br>322 - 336 -<br>938 - 952 -<br>872 - 886 -
	(a) Gorai.002G177000	DELLA protein (AtRGA)	GRAS	GaBFT-L1<br>GhBFT-L1-A<br>GhBFT-L1-D<br>GrBFT-L1	1089 - 1108 -<br>637 - 656 -<br>1577 - 1596 -<br>1510 - 1529 -
	(a) Gorai.001G036500	ERF003 (AtETHYLENE AND SALT INDUCEIBLE3)	ERF	GaBFT-L1<br>GhBFT-L1-A<br>GhBFT-L1-D<br>GrBFT-L1	1089 - 1108 -<br>637 - 656 -<br>1577 - 1596 -<br>1510 - 1529 -
BFT-L2	(a) Gorai.011G238900	AUXIN RESPONSE FACTOR10-related (AtARF16)	Arf	GaBFT-L2<br>GhBFT-L2-A<br>GhBFT-L2-D<br>GrBFT-L2	71 - 91 +<br>138 - 158 +<br>341 - 361 +<br>341 - 361 +
	(a) Gorai.009G271100<br>(b) Gorai.013G096100	APETELLA3 (AtAP3)<br>AG-like AGL8-related (AtAP1)	MIKC-MADS	GaBFT-L2<br>GhBFT-L2-A<br>GhBFT-L2-D<br>GrBFT-L2	976 - 990 -<br>986 - 1000 -<br>1115 - 1126 -<br>1115 - 1126 -
	(a) Gorai.004G186700<br>(b) Gorai.007G112500	(AtNAC071)<br>NAC-43-related (AtNac SECONDARY WALL THICKENING1)	NAC	GaBFT-L2<br>GhBFT-L2-A<br>GhBFT-L2-D	1249 - 1263 -<br>1257 - 1271 -<br>1361 - 1375 -

				GrBFT-L2	1361 - 1375 -
	(a) Gorai.008G218300	Beta Amylase 8 (AtBeta Amylase 8)	BES1	GaBFT-L2<br>GhBFT-L2-A<br>GhBFT-L2-D<br>GrBFT-L2	1545 - 1559 +<br>1553 - 1567 +<br>1548 - 1562 +<br>1548 - 1562 +
MFT-L1	(a) Gorai.007G274700<br>(b) Gorai.008G185500	MYB-related (AtPhytochrome-Dependent Late-Flowering)<br>MYB-related (AtHHO2)	G2-like	GaMFT-L1<br>GhMFT-L1-A<br>GhMFT-L1-D<br>GrMFT-L1	993(5) - 1005(9) +<br>1000(2) - 1012(16) +<br>1005(7) - 1017(21) +<br>1027(29) - 1039(43) +
	(a) Gorai.004G267300<br>(b) Gorai.005G195300<br>(c) Gorai.006G060900<br>(d) Gorai.007G188800<br>(e) Gorai.008G155200<br>(f) Gorai.008G236400<br>(g) Gorai.009G186000<br>(h) Gorai.011G090000	BEARSKIN1 (AtBEARSKIN2)<br>No Apical Meristem (NAM) (AtVND INTERACTING2)<br>NAM(AtNAC058)<br>(AtNAC028)<br>NAC protein 10 (AtNacSECONDARY WALL THICKENING1)<br>NAC26-related (AtVND4)<br>CUP SHAPE COTYLEDON 3 (AtCUC3)<br>NAM (AtLOV1)	NAC	GaMFT-L1<br>GhMFT-L1-A<br>GhMFT-L1-D<br>GrMFT-L1	1126(29) -<br>1144(49) -<br>1132 (36) -<br>1150(55) -<br>1134(37) -<br>1152(57) -<br>1156(59) -<br>1172(79) -
	(a) Gorai.005G195300<br>(b) Gorai.009G166300<br>(c) Gorai.010G124100<br>(d) Gorai.013G146300	NAM/NAC10-related (AtVNI2)<br>NAM/NAC10 (AtSND3)<br>NAC38-related (AtNAC038)<br>NAC20-related (AtNAC20)	NAC	GaMFT-L1<br>GhMFT-L1-A<br>GhMFT-L1-D<br>GrMFT-L1	1129(30) - 1144(9) +<br>1135(6) - 1150(5) +<br>1137(8) - 1152(7) +<br>1159(60) - 1174(9) +

	(a) Gorai.005G015900<br>(b) Gorai.011G209200	(AtbZIP69)<br>bZIP-1 (AtbZIP18)	bZIP	GaMFT-L1<br>GhMFT-L1-A<br>GhMFT-L1-D<br>GrMFT-L1	1239 - 1249 -<br>1245 - 1255 -<br>1247 - 1257 -<br>1269 - 1279 -
	(a) Gorai.005G015900<br>(b) Gorai.009G285000	(AtbZIP69)<br>VIRE2 INTERACTING PROTEIN1<br>(AtVIP1)	bZIP	GaMFT-L1<br>GhMFT-L1-A<br>GhMFT-L1-D<br>GrMFT-L1	1238(40) - 1249(50) +<br>1244(6) - 1255(6) +<br>1246(8) - 1257(8) +<br>1268(70) - 1279(80) +
	(a) Gorai.003G021000<br>(b) Gorai.009G100100<br>(c) Gorai.009G319300	Dof 3.4 (At OBF BINDING PROTEIN 1)<br>(At OBF BINDING PROTEIN 3)<br>Dof2.4 (AtDof2.4)	Dof	GaMFT-L1<br>GhMFT-L1-A<br>GhMFT-L1-D	1792(3) - 1812(3) -<br>1793(4) - 1813(4) -<br>1791(3) - 1811(3) -
	(a) Gorai.005G015900	(AtbZIP69)	bZIP	GaMFT-L1<br>GhMFT-L1-A<br>GhMFT-L1-D<br>GrMFT-L1	1825 - 1835 +<br>1826 - 1836 +<br>1825 - 1835 +<br>1825 - 1835 +
	(a) Gorai.005G188700	sterol regulatory protein (AtBIG PETAL)	bHLH	GaMFT-L1<br>GhMFT-L1-A<br>GhMFT-L1-D<br>GrMFT-L1	1836 - 1856 +<br>1837 - 1857 +<br>1836 - 1856 +<br>1836 - 1856 +
	(a) Gorai.009G128000	GENERAL TF IIIA (AtTFIIIA)	C2H2	GaMFT-L1<br>GhMFT-L1-A<br>GhMFT-L1-D<br>GrMFT-L1	1913 - 1931 +<br>1914 - 1932 +<br>1913 - 1931 +<br>1913 - 1931 +
	(a) Gorai.009G128000	GENERAL TF IIIA (AtTFIIIA)	C2H2	GaMFT-L1	1974 - 1992 +

				GhMFT-L1-A<br>GhMFT-L1-D<br>GrMFT-L1	1975 - 1993 +<br>1974 - 1992 +<br>1974 - 1992 +
	(a) Gorai.009G128000	GENERAL TF IIIA (AtTFIIIA)	C2H2	GaMFT-L1<br>GhMFT-L1-A<br>GhMFT-L1-D<br>GrMFT-L1	1977 - 1995 +<br>1978 - 1996 +<br>1975 - 1995 +<br>1977 - 1995 +
	(a) Gorai.009G128000<br>(b) Gorai.010G025200	GTFIIIA (AtTFIIIA)<br>ZINC FINGER PROTEIN3-related (AtSALT TOLERANCE ZINC FINGER)	C2H2	GaMFT-L1<br>GhMFT-L1-A<br>GhMFT-L1-D<br>GrMFT-L1	1979 - 1989(98) +<br>1980(1) - 1990(9) +<br>1979(80) - 1989(98) +<br>1979(80) - 1989(98) +
	(a) Gorai.011G234400	C2H2-related	C2H2	GaMFT-L1<br>GhMFT-L1-A<br>GhMFT-L1-D<br>GrMFT-L1	1979 - 1989 -<br>1980 - 1990 -<br>1979 - 1989 -<br>1979 - 1989 -
MFT-L2	(a) Gorai.005G211900<br>(b) Gorai.013G068600	TCP21-related (AtTCP7)<br>TCP21-related (AtTCP7)	TCP	GaMFT-L2<br>GhMFT-L2-A<br>GhMFT-L2-D<br>GrMFT-L2	1010 - 1020 -<br>1016 - 1026 -<br>1007 - 1017 -<br>1008 - 1018 -
	(a) Gorai.006G043800<br>(b) Gorai.006G197000	TCP20-related (AtTCP20)<br>TCP19 (AtTCP19)	TCP	GaMFT-L2<br>GhMFT-L2-A<br>GhMFT-L2-D<br>GrMFT-L2	1013 - 1020 +<br>1019 - 1026 +<br>1010 - 1017 +<br>1011 - 1018 +
	(a) Gorai.009G319300	Dof2.4 (AtDof2.4)	Dof	GaMFT-L2<br>GhMFT-L2-A<br>GhMFT-L2-D<br>GrMFT-L2	1299 - 1319 -<br>739 - 759 -<br>1296 - 1316 -<br>1297 - 1317 -

	(a) Gorai.006G188900	(AtDof5.6)	Dof	GaMFT-L2<br>GhMFT-L2-A<br>GhMFT-L2-D<br>GrMFT-L2	1301 - 1321 +<br>1301 - 1321 +<br>209 - 229 -<br>253 - 273 -
	(a) Gorai.007G051100<br>(b) Gorai.007G206000	ATHHB-21-related (AtHB40)<br>ATHHB-21-related (AtHB40)	HD-ZIP	GaMFT-L2<br>GhMFT-L2-Ap<br>GhMFT-L2-D<br>GrMFT-L2	1399 - 1419 +<br>1399 - 1419 +<br>1399 - 1419 +<br>1399 - 1419 +
	(a) Gorai.001G199200	WUSCHEL-related homeobox 10-related (AtWOX13)	WOX	GaMFT-L2<br>GhMFT-L2-A<br>GhMFT-L2-D<br>GrMFT-L2	1516 - 1525 +<br>1516 - 1525 +<br>1516 - 1525 +<br>1516 - 1525 +
	(a) Gorai.001G087400	MYB113-related (AtMYB113)	MYB	GaMFT-L2<br>GhMFT-L2-A<br>GhMFT-L2-D<br>GrMFT-L2	1760 - 1770 -<br>1760 - 1770 -<br>1760 - 1770 -<br>1760 - 1770 -
	(a) Gorai.009G301100	(AtMYB33)	MYB	GaMFT-L2<br>GhMFT-L2-A<br>GhMFT-L2-D<br>GrMFT-L2	1760 - 1770 -<br>1760 - 1770 -<br>1760 - 1770 -<br>1760 - 1770 -
	(a) Gorai.009G301400	abscisic acid 5-like related 2 (AtAREB3)	bZIP	GaMFT-L2<br>GhMFT-L2-A<br>GhMFT-L2-D<br>GrMFT-L2	1781 - 1795 +<br>1781 - 1795 +<br>1781 - 1795 +<br>1781 - 1795 +
	(a) Gorai.001G183400	PIF4-related (AtPIF1)	bHLH	GaMFT-L2<br>GhMFT-L2-A<br>GhMFT-L2-D<br>GrMFT-L2	1786 - 1799 -<br>1786 - 1799 -<br>1786 - 1799 -<br>1786 - 1799 -
	(a) Gorai.008G024700	abscisic acid 5-like related (AtABF1)	bZIP	GaMFT-L2	1786 - 1793 +

				GhMFT-L2-A	1786 - 1793 +
				GhMFT-L2-D	1786 - 1793 +
				GrMFT-L2	1786 - 1793 +
	(a) Gorai.008G024700<br>(b) Gorai.009G212600	abscisic acid 5-like related (AtABF1) camp-response element binding protein related (AtABF2)	bZIP	GaMFT-L2	1783(6) - 1793(1800) -
				GhMFT-L2-A	1783(6) - 1793(1800) -
				GhMFT-L2-D	1783(6) - 1793(1800) -
				GrMFT-L2	1783(6) - 1793(1800) -
	(a) Gorai.008G218300	Beta Amylase 8 (AtBAM8)	BES1	GaMFT-L2	1784 - 1798 +
				GhMFT-L2-A	1784 - 1798 +
				GhMFT-L2-D	1784 - 1798 +
				GrMFT-L2	1784 - 1798 +
	(a) Gorai.007G205700	G-box binding factor 1 (AtGBF1)	bZIP	GaMFT-L2	1853 - 1862 +
				GhMFT-L2-A	1853 - 1862 +
				GhMFT-L2-D	1853 - 1862 +
				GrMFT-L2	1853 - 1862 +
	(a) Gorai.010G025200	ZINC FINGER PROTEIN3-related (AtSALT TOLERANCE ZINC FINGER	C2H2	GaMFT-L2	1878 - 1888 +
				GhMFT-L2-A	1878 - 1888 +
				GhMFT-L2-D	1878 - 1888 +
				GrMFT-L2	1878 - 1888 +
	(a) Gorai.007G371500	aspartate kinase	Trihelix	GaMFT-L2	1883 - 1896 -
				GhMFT-L2-A	1883 - 1896 -
				GhMFT-L2-D	1883 - 1896 -
				GrMFT-L2	1883 - 1896 -

CETS promoters from *G. raimondii*, *G. arboreum,* and *G. hirsutum* genomes were used as input to predict conserved transcription factors using a *G. raimondii* transcription factor database. Descriptions of transcription factors were mined from Phytozome (https://phytozome.jgi.doe.gov/pz/portal.html). The closest Arabidopsis homologs were identified from BLAST queries; homologs

in red font indicate orthologous genes based upon reciprocal BLASTp searches. Binding sites reflect distance in nucleotides from the 5’ promoter; strand binding is shown as “+” or “-”.

```
PpMFTL3         -----------------------------------------------------MSRSVDP   7
PpMFTL4         -----------------------------------------------------MARSIDP   7
PpMFTL1         -----------------------------------------------------MPRSIDP   7
PpMFTL2         -----------------------------------------------------MARSIDP   7
SlMFT           -----------------------------------------------------MGGKVDP   7
AtMFT           -----------------------------------------------------MAASVDP   7
CcMFT-L3        -----------------------------------------------------MAVSVDP   7
CoMFT-L1        -----------------------------------------------------MAVSVDP   7
CcMFT-L1        -----------------------------------------------------MAVSVDP   7
TcMFT-L1        MPYAWHIRHTNLSHFSPFTPLYKLLLAFLYSTLTPNQTNILISSPLLFALFFSMAVSVDP   60
GaMFT-L1        -----------------------------------------------------MAASVDP   7
GrMFT-L1        -----------------------------------------------------MAASVDP   7
GhMFT-L1-Dt     -----------------------------------------------------MAASVDP   7
GhMFT-L1-At     -----------------------------------------------------MAASVDP   7
SlSP2G          --------------------------------------------------METSARSVDP   10
CoMFT-L2        -----------------------------------------------------MARSVEP   7
CcMFT-L2        -----------------------------------------------------MARSVEP   7
TcMFT-L2        -----------------------------------------------------MARSVEP   7
GrMFT-L2        -----------------------------------------------------MARSVEP   7
GhMFT-L2-Dt     -----------------------------------------------------MARSVEP   7
GaMFT-L2        -----------------------------------------------------MARSVEP   7
GhMFT-L2-At     -----------------------------------------------------MARSVEP   7
SlSP5G          -------------------------------------------------------MPRDP   5
```

```
SlSP11D     -----------------------------------------------------MQRERDT      7
AtFT        ----------------------------------------------------MSINIRDP      8
AtTSF       ----------------------------------------------------MSLSRRDP      8
SpSP11C     ---------------------------------------------------MSSIRGRDT      9
SpSP6A      ------------------------------------------------------MPRVDP      6
SlSP3D      -----------------------------------------------------MPRERDP      7
CoSFT       -----------------------------------------------------MPRDRDP      7
CcSFT       -----------------------------------------------------MPRDRDP      7
TcSFT       -----------------------------------------------------MPRERDP      7
GaSFT       -----------------------------------------------------MPRDRDP      7
GhSFT-At    -----------------------------------------------------MPRDRDP      7
GrSFT       -----------------------------------------------------MPRDRDP      7
GhSFT-Dt    -----------------------------------------------------MPRDRDP      7
SlSP        ----------------------------------------------------MASKMCEP      8
AtCEN       ----------------------------------------------------MARISSDP      8
CoSP        -----------------------------------------------------MAKLSDP      7
CcSP        -----------------------------------------------------MAKLSDP      7
TcSP        -----------------------------------------------------MAKLSDP      7
GrSP        -----------------------------------------------------MAKLSDP      7
GhSP-Dt     -----------------------------------------------------MAKLSDP      7
GaSP        -----------------------------------------------------MAKLSDP      7
GhSP-At     -----------------------------------------------------MAKLSDP      7
AtTFL1      -------------------------------------------------MENMGTRVIEP      11
SlSP9D      -----------------------------------------------------MARSLEP      7
```

```
GaTFL1-L2        --------------------------------------------------------MGEP        4
GhTFL1-L2-At     --------------------------------------------------------MGEP        4
GrTFL1-L2        --------------------------------------------------------MGEP        4
GhTFL1-L2-Dt     --------------------------------------------------------MGEP        4
GrTFL1-L1        -----------------------------------------------------MAREVEP        7
GhTFL1-L1-Dt     -----------------------------------------------------MAREVEP        7
GaTFL1-L1        -----------------------------------------------------MAREVEP        7
GhTFL1-L1-At     -----------------------------------------------------MAREVEP        7
TcTFL1           -----------------------------------------------------MSRAAEP        7
CoTFL1           ----------------------------------------------------MSTRSIEP        8
CcTFL1           ----------------------------------------------------MSTRSIEP        8
AtBFT            -----------------------------------------------------MSREIEP        7
SlBFT-L1         ----------------------------------------------------MSCRDIEP        8
SlBFT-L2         ----------------------------------------------------MSCRDIEP        8
SlBFT-L3         ---------------------------------------------------MSSRSTCEP        9
TcBFT            -----------------------------------------------------MSRVPEP        7
GaBFT-L2         -----------------------------------------------------MSRVPEP        7
GhBFT-L2-At      -----------------------------------------------------MSRVPEP        7
GrBFT-L2         -----------------------------------------------------MSRVPEP        7
GhBFT-L2-Dt      -----------------------------------------------------MSRVPEP        7
GhBFT-L1-Dt      -----------------------------------------------------MSRVPDP        7
GrBFT-L1         -----------------------------------------------------MSRVPDP        7
GaBFT-L1         -----------------------------------------------------MSRVPDP        7
GhBFT-L1-At      -----------------------------------------------------MSRVPDP        7
```

```
CoBFT            ----------------------------------------------------MSRSVLEP     8
CcBFT            ----------------------------------------------------MSRSVHEP     8
                                                                           :

PpMFTL3          LVVGRVIGVVIDMFAPSV--DMAVVYTSRKVS-NGCQMKPSATNEAPTVHVTGNNG-DNN     63
PpMFTL4          LVVGKVIGDVIDTFVPSV--DMAIHYSTRQVT-NGCQMMPSATAQAPEIHLSDKSG-GNN     63
PpMFTL1          LIVGKVIGDVIDTFVPRV--DMAIHYSTRQVT-NGCQLKPSATAQAPEIQLSDKSG-DNN     63
PpMFTL2          LVVGKVIGDVIDTFVPSV--DMAIHYSSRQVT-NGCQMKPSATAQAPEIQLSDNSE-GNN     63
SlMFT            LVVGRVIGDVVDMFVPSV--TMSVHYANKHVN-NGCDIKPSIATEPPKIAIGGQPD---E     61
AtMFT            LVVGRVIGDVLDMFIPTA--NMSVYFGPKHIT-NGCEIKPSTAVNPPKVNISGHSD---E     61
CcMFT-L3         LVVGRVIGDVVDMFVPTV--TTSIYYASKHVT-NGCHVKPSIAINPPKVSIDGHPG---H     61
CoMFT-L1         LVVGRVIGDVVDMFVPTV--TMSIYYASKHVT-NGCDVKPSMAINPPKVSIDGHPD---H     61
CcMFT-L1         LVVGRVIGDVVDMFVPSV--TMSIYYASKHVT-NGCDVKPSMAINPPKVSIDGHPD---H     61
TcMFT-L1         LVVGRVIGDVVDMFVPTV--TMSVYYGSRHVT-NGCDIKPSTTINPPKVSINGHSD---E     114
GaMFT-L1         LVVGRVIGDVVDMFVPTV--TMSVYYGSKHVS-NGCDIKPSMAINPPKVAIDGLPD---Q     61
GrMFT-L1         LVVGRVIGDVVDMFVPTV--TMSVYYGSKHVS-NGCDIKPSMAINPPKVAIDGLPD---Q     61
GhMFT-L1-Dt      LVVGRVIGDVVDMFVPTV--TMSVYYGSKHVS-NGCDIKPSMAINPPKVAIDGLPD---Q     61
GhMFT-L1-At      LVVGRVIGDVVDMFVPTV--TMSVYYGSKHVS-NGCDIKPSMAINPPKVAIDGLPD---Q     61
SlSP2G           LVVGKVIGDVLDMFVPVV--DFTVEYASKQISNNGVEIKPAEAAQKPRVHIKGSLH-SNN     67
CoMFT-L2         LVVGRVIGDVLDMFTPAA-TVFTAHYGSKQ------------------------------     36
CcMFT-L2         LVVGRVIGDVLDMFTPAAATVFTAHYGSKQVT-NGCDIKPSAASDKPHAQILGPPDNSTG     66
TcMFT-L2         LVVGRVIGDVLDIFTPAA--ELTVHYSTKQVH-NGCDIKPSSAADKPHVRILSPVV-SSS     63
GrMFT-L2         LVVGRVIGDVLDMFTPAS--EFIVRYGTKQVT-NGCDIKPSAAADKPHVQILGHPF-SSN     63
GhMFT-L2-Dt      LVVGRVIGDVLDMFTPAS--EFTVRYGTKQVT-NGCDIKPSAAADKPHVQILGHPF-SSN     63
```

```
GaMFT-L2        LVVGRVIGDVLDMFTPAS--EFTVRYGTKQVT-NGCDIKPSAAADKPHVQILGHPF-SSN    63
GhMFT-L2-At     LVVGRVIGDVLDMFTPAS--EFTVRYGTKQVT-NGCDIKPSAAADKPHVQILGHPF-SSN    63
SlSP5G          LIVSGVVGDVVDPFTRCV--DFGVVYN-NRVVYNGCSLRPSQVVNQPRVDIDGDDL--RT    60
SlSP11D         LRLARVIGDVLDPFTKSI--NLRVVYN-NKEIRNGCDLRPSMVVNQPRVEVGGDDF--QT    62
AtFT            LIVSRVVGDVLDPFNRSI--TLKVTYG-QREVTNGLDLRPSQVQNKPRVEIGGEDL--RN    63
AtTSF           LVVGSVVGDVLDPFTRLV--SLKVTYG-HREVTNGLDLRPSQVLNKPIVEIGGDDF--RN    63
SpSP11C         LELGGVISDVLDPFTRSI--NLSVVYN-HREVINGTNLRPSQITNQPRVEVGGNDL--ST    64
SpSP6A          LIVGRVIGEVLDPFTRSV--DLRVVYN-NREVNNACVLKPSQVVMQPKVYIGGDDL--RT    61
SlSP3D          LVVGRVVGDVLDPFTRTI--GLRVIYR-DREVNNGCELRPSQVINQPRVEVGGDDL--RT    62
CoSFT           LVVGRVIGDVLDPFTRSI--SLRVSFG-GREVNNGCELKPSQVVNQPRVDIGGEDL--RT    62
CcSFT           LVVGRVIGDVLDPFTRSI--SLRVSFG-GREVNNGCELKPSQVVNQPRVDIGGEDL--RT    62
TcSFT           LVVGRVIGDVLDPFTRSI--SLRVTFA-CREVNNGCELKPSQVVNQPRVDIGGDDL--RT    62
GaSFT           LVVGRVIGDVLDPFTRSI--SLRVTYA-TRDVNNGVELKPSQVVNQPRVDIGGDDL--RT    62
GhSFT-At        LVVGRVIGDVLDPFTRSI--SLRVTYA-TRDVNNGVELKPSQVVNQPRVDIGGDDL--RT    62
GrSFT           LVVGRVIGDVLDPFTRSI--SLRVTYA-TRDVSNGVELKPSQVVNQPRVDIGGDDL--RT    62
GhSFT-Dt        LVVGRVIGDVLDPFTRSI--SLRVTYA-TRDVSNGVELKPSQVVNQPRVDIGGDDL--RT    62
SlSP            LVIGRVIGEVVDYFCPSV--KMSVVYNNNKHVYNGHEFFPSSVTSKPRVEVHGGDL--RS    64
AtCEN           LMVGRVIGDVVDNCLQAV--KMTVTYNSDKQVYNGHELFPSVVTYKPKVEVHGGDM--RS    64
CoSP            LVVGRVIGDVVDAINPCV--KITVTFNSNKQVYNGHEFFPSSVTTKPK------------    53
CcSP            LVVGRVIGDVVDAINPCV--KITVTFNSNKQVYNGHEFFPSSVTTKPK------------    53
TcSP            LVVGRVIGDVIDAITPSV--KMTVTFNANKQVYNGHELFPSSVTNKPKVDVHGGDM--RS    63
GrSP            LVLGRVIGDVIDALSPSV--KMSVTFNTNKQVYNGHEFFPSAVTNKPKVEVHGGDM--RS    63
GhSP-Dt         LVLGRVIGDVIDALSPSV--KMSVTFNTNKQVYNGHEFFPSAVTNKPKVEVHGGDM--RS    63
GaSP            LVVGRVIGDVIDALSPSV--KMSVTFNTNKQVYNGHEFFPSAVTNKPKVEVHGGDM--RS    63
```

GhSP-At	LVVGRVIGDVIDALSPSV--KMSVTFNTNKQVYNGHEFFPSAVTNKPKVEVHGGDM--RS	63
AtTFL1	LIMGRVVGDVLDFFTPTT--KMNVSYN-KKQVSNGHELFPSSVSSKPRVEIHGGDL--RS	66
SlSP9D	LIVGRVIGDVIDSFNPTI--KMSITYN-NKLVCNGHELFPSVVSSRPKVEVQGGDL--RT	62
GaTFL1-L2	LIVGGVVGDVLDSFNPSI--KMSVTFN-NKQVFNGHEFYPSSVATKPRVEIQGGDL--RT	59
GhTFL1-L2-At	LIVGGVVGDVLDSFNPSI--KMSVTFN-NKQVFNGHEFYPSSVATKPRVEIQGGDL--RT	59
GrTFL1-L2	LIVGGVIGDVLDSFNPSI--KMSVTFN-NKQVFNGHEFYPSSVATKPRVEIQGGDL--RT	59
GhTFL1-L2-Dt	LIVGGVIGDVLDSFNPSI--KMSVTFN-NKQVFNGHEFYPSSVATKPRVEIQGGDL--RT	59
GrTFL1-L1	LMVGRVIGDVMDSFIPSI--KMLVTFN-NKQVFNGHEFYPSTVVTKPRVEVAGGDM--RT	62
GhTFL1-L1-Dt	LMVGRVIGDVMDSFIPSI--KMLVTFN-NKQVFNGHEFYPSTVVTKPRVEVAGGDM--RT	62
GaTFL1-L1	LMVGRVIGDVMDSFIPSI--KMSVTFN-NKQVFNGHEFYPSTVVTKPRVEVVGGDM--RT	62
GhTFL1-L1-At	LMVGRVIGDVMDSFIPSI--KMSVTFN-NKQVFNGHEFYPSTVVTKPRVEVVGGDM--RT	62
TcTFL1	LVVGRVIGDVLDSFIPSI--TMTVTFN-NKRVFNGHEFYPSTVATKPRVEIEGGDM--RT	62
CoTFL1	LIVGRVIGDVLDSFIPSI--IMTVSFN-NKKVFNGHEFFPSTVASRPRVEIEGGDL--RT	63
CcTFL1	LIVGRVIGDVLDSFIPSI--TMTASFN-NKKVFNGHEFFPSTVAFRPRVEIEGGDL--RT	63
AtBFT	LIVGRVIGDVLEMFNPSV--TMRVTFNSNTIVSNGHELAPSLLLSKPRVEIGGQDL--RS	63
SlBFT-L1	LIVAKVIGEVVDSFNPSV--KMNVTYNGTKQVFNGHELMPLVIASKPRVEIGGEDM--RS	64
SlBFT-L2	LIVARVIGEVVDSFNPSV--KMNVIYNGTKQVFNGHELMPLVIASKPRVEIGGEDM--RS	64
SlBFT-L3	LAVGRVIGEVVDSFSPSV--KMKVIYNGRKQVSNGHEIMPAVVATQPRVEIGGEDM--RS	65
TcBFT	LTVGRVIGEVVDNFTPSV--KMTVTYNSNKQVANGHELMPAVIVARPRVEIGGEDM--RA	63
GaBFT-L2	LTVGRVIGEVVDNFTQSV--QMTVTYNPNKQVANGHELMPAVISARPRVEIGGNDM--RD	63
GhBFT-L2-At	LTVGRVIGEVVDNFTQSV--QMTVTYNPNKQVANGHELMPAVISARPRVEIGGNDM--RD	63
GrBFT-L2	LTVGRVIGEVVDNFTPSV--QMTVTYNPNKQVANGHELMPAAISARPRVEIGGNDM--RD	63
GhBFT-L2-Dt	LTVGRVIGEVVDNFTPSV--QMTVTYNPNKQVANGHELMPAAISARPRVEIGGNDM--RD	63
GhBFT-L1-Dt	LIIGRVIGEVVDNFFPSV--KITVTYNSNKQVANGHELMPALITGRPRVEIGGDDM--RP	63

```
GrBFT-L1          LIIGRVIGEVVDNFFPSV--KITVTYNSNKQVANGHELMPALITGRPRVEIGGDDM--RP    63
GaBFT-L1          LIIGRVIGEVVDNFFPSV--KITVTYNSNKQVANGHELMPALITARPRVEIGGDDM--RP    63
GhBFT-L1-At       LIIGRVIGEVVDNFFPSV--KITVTYNSNKQVANGHELMPALITARPRVEIGGDDM--RP    63
CoBFT             LSIGRVIGEVVDYFTPSV--KLIVTYNSNKQVANGHELMPALISARPRVEIGGDDL--RS    64
CcBFT             LSIGRVIGEVVDYFTPSV--KLIVTYNSNKQVANGHELMPAVISARPRVEIGGDDM--RA    64
                  * :. *:. *::             :

PpMFTL3           FFTLIMTDPDAPSPSEPSLREWVHW------------IVTDIPGNSSTTTSGQGSKRARE    111
PpMFTL4           LYTLIMIDPDAPSPSEPTLREWLHW------------IVTDIPGNSGGSEMTSGFPRLNE    111
PpMFTL1           YYTLVMTDPDAPSPSEPSLREWLHW------------IVTDIPGNSGGSETNTGFPWLSE    111
PpMFTL2           YYTLIMTDPDAPSPSEPSLREWLHW------------IVTDIPGNSGGSETTSGFSWLQE    111
SlMFT             FYTLVMTDPDAPSPSEPTMREWVHW------------IVTDIPGCSN-------------    96
AtMFT             LYTLVMTDPDAPSPSEPNMREWVHW------------IVVDIPGGTN-------------    96
CcMFT-L3          LYTLVMTDPDAPSPSEPSMREWVHW------------KGDT-------------------    90
CoMFT-L1          LYTLVMTDPDAPSPSEPSMREWVHW------------IVCDIPGGTN-------------    96
CcMFT-L1          LYTLVMTDPDAPSPSEPSMREWVHW------------IVCDIPGGTN-------------    96
TcMFT-L1          LYTLVMTDPDAPSPSEPSMREWVHW------------IVSDIPGGTN-------------    149
GaMFT-L1          FYTLVMTDPDAPSPSEPTMREWVHW------------IVSDIPGGTN-------------    96
GrMFT-L1          FYTLVMTDPDAPSPSEPTMREWVHW------------IVSDIPGGTN-------------    96
GhMFT-L1-Dt       FYTLVMTDPDAPSPSEPTMREWVHW------------IVSDIPGGTN-------------    96
GhMFT-L1-At       FYTLVMTDPDAPSPSEPTMREWVHW------------IVSDIPGGTN-------------    96
SlSP2G            LYTLVMADPDAPSPSEPTFREWLHW------------IVTDIPEGGD-------------    102
CoMFT-L2          ----VMVDPDAPSPSEPRLRE----------------IVVDIPHGHD-------------    63
CcMFT-L2          LYTLVMVDPDAPSPSEPRLREWLHW------------IVVDIPHGHD-------------    101
```

```
TcMFT-L2        LYTLVMVDPDAPTPSEPRLREWLHW------------IVVDIPEGHD-------------    98
GrMFT-L2        LYTLVMVDPDAPSPSEPRLREWLHW------------IVVDVPEGQD-------------    98
GhMFT-L2-Dt     LYTLVMVDPDAPSPSEPRLREWLHW------------IVVDVPEGQD-------------    98
GaMFT-L2        LYTLVMVDPDAPSPSEPRLREWLHW------------IVVDIPEGQD-------------    98
GhMFT-L2-At     LYTLVMVDPDAPSPSEPRLREWLHW------------IVVDIPEGQD-------------    98
SlSP5G          FYTLIMVDPDAPNPSNPNLREYLHW------------LVTDIPAATG-------------    95
SlSP11D         FYTLVMVDPDAPTPSNPCHKDYLHW------------LVTNIPASTG-------------    97
AtFT            FYTLVMVDPDVPSPSNPHLREYLHW------------LVTDIPATTG-------------    98
AtTSF           FYTLVMVDPDVPSPSNPHQREYLHW------------LVTDIPATTG-------------    98
SpSP11C         FYTLIVVDPDAPSPSNPNLREYLHW------------LVTDIPATTG-------------    99
SpSP6A          FYTLIMVDPDAPSPSNPNLREYLHW------------LVTDIPATTD-------------    96
SlSP3D          FFTLVMVDPDAPSPSDPNLREYLHW------------LVTDIPATTG-------------    97
CoSFT           FYTLVMVDPDAPSPSDPNLREYLHW------------LVTDIPATTG-------------    97
CcSFT           FYTLVMVDPDAPSPSDPNLREYLHW------------LVTDIPATTG-------------    97
TcSFT           FYTLVMVDPDAPSPSDPNLREYLHW------------LVTDIPATTG-------------    97
GaSFT           FYTLVMVDPDAPSPSDPNLREYLHW------------LVTDIPATTG-------------    97
GhSFT-At        FYTLVMVDPDAPSPSDPNLREYLHW------------LVTDIPATTG-------------    97
GrSFT           FYTLVMVDPDAPSPSDPNLREYLHW------------LVTDIPATTG-------------    97
GhSFT-Dt        FYTLVMVDPDAPSPSDPNLREYLHW------------LVTDIPATTG-------------    97
SlSP            FFTLIMIDPDVPGPSDPYLREHLHW------------IVTDIPGTTD-------------    99
AtCEN           FFTLVMTDPDVPGPSDPYLREHLHW------------IVTDIPGTTD-------------    99
CoSP            ----VMTDPDVPGPSDPYLKEHLHW------------IVTDIPGTTD-------------    84
CcSP            ----VMTDPDVPGPSDPYLKEHLHW------------IVTDIPGTTD-------------    84
TcSP            FFTLVMTDPDVPGPSDPYLREHLHW------------IVTDIPGTTD-------------    98
```

GrSP	FFTLVMTDPDVPGPSDPYLREHLHW------------IVTDIPGTTD-------------	98
GhSP-Dt	FFTLVMTDPDVPGPSDPYLREHLHW------------IVTDIPGTTD-------------	98
GaSP	FFTLVMTDPDVPGPSDPYLREHLHW------------IVTDIPGTTD-------------	98
GhSP-At	FFTLVMTDPDVPGPSDPYLREHLHW------------IVTDIPGTTD-------------	98
AtTFL1	FFTLVMIDPDVPGPSDPFLKEHLHW------------IVTNIPGTTD-------------	101
SlSP9D	FFTLVMTDPDVPGPSDPYMREHLHW------------IITDIPGTTD-------------	97
GaTFL1-L2	FFTLVMTDPDVPGPSDPYLREHLHW------------IVTDIPGTTD-------------	94
GhTFL1-L2-At	FFTLVMTDPDVPGPSDPYLREHLHW------------IVTDIPGTTD-------------	94
GrTFL1-L2	FFTLVMTDPDVPGPSDPYLREHLHW------------IVTDIPGTTD-------------	94
GhTFL1-L2-Dt	FFTLVMTDPDVPGPSDPYLREHLHW------------IVTDIPGTTD-------------	94
GrTFL1-L1	FFTLVMTDPDVPGPSDPYLREHLHW------------IVTDIPGTTD-------------	97
GhTFL1-L1-Dt	FFTLVMTDPDVPGPSDPYLREHLHW------------IVTDIPGTTD-------------	97
GaTFL1-L1	FFTLVMTDPDVPGPSDPYLREHLHW------------IVTDIPGTTD-------------	97
GhTFL1-L1-At	FFTLVMTDPDVPGPSDPYLREHLHW------------IVTDIPGTTD-------------	97
TcTFL1	FFTLVMTDPDVPGPSDPYLREHIHW------------IVTDIPGTTD-------------	97
CoTFL1	FFTLVMTDPDVPGPSDPYLREHLHW------------IVTDIPGTTD-------------	98
CcTFL1	FFTLVMTDPDVPGPSDPYLREHLHW------------IVTDIPGTTD-------------	98
AtBFT	FFTLIMMDPDAPSPSNPYMREYLHW------------MVTDIPGTTD-------------	98
SlBFT-L1	AYTLVMIDPDVPGPSDPYLREHLHW------------IVTDIPGSTD-------------	99
SlBFT-L2	AYTLIMTDPDVPGPSDPYLREHLHW------------IVTDIPGSTD-------------	99
SlBFT-L3	AYTLIMTDPDAPSPSDPYLREHLHW------------IVTDIPGTTD-------------	100
TcBFT	AYTLILTDPDAPSPSDPYLREHLHCPQSKLQTNISSRMVTDIPGTTD-------------	110
GaBFT-L2	AYTLIMTDPDAPSPSGPFLREHLHW------------MVTDVPGTTD-------------	98
GhBFT-L2-At	AYTLIMTDPDAPSPSGPFLREHLHW------------MVTDVPGTTD-------------	98

```
GrBFT-L2          AYTLVMTDPDAPSPSDPFLREHLHW------------MVTDVPGTTD-------------    98
GhBFT-L2-Dt       AYTLIMTDPDAPSPSDPFLREHLHW------------MVTDVPGTTD-------------    98
GhBFT-L1-Dt       SYTLIMTDPDAPSPSDLYLREHLHW------------MVTDIPGTTD-------------    98
GrBFT-L1          SYTLVMTDPDAPSPSDPYLIEHLHW------------MVTDIPGTTD-------------    98
GaBFT-L1          SYTLIMTDPDAPSPSDPYLREHLHW------------MVTDIPGTTD-------------    98
GhBFT-L1-At       CYTLIMTDPDAPSPSDPYLREHLHW------------MVTDIPGTTD-------------    98
CoBFT             AYTLIMTDPDAPSPSDPYLREHLHW------------MVTDIPGTTD-------------    99
CcBFT             AYTLIMTDPDAPSPSDPYLREHLHW------------MVTDIPGTTD-------------    99
                      :: ***.* **     :

PpMFTL3           PASSAKQPNVERKKKGPAASTTDKELPSAADQGAAKPRTSGKEVVPYVGPCPPIGIHRYI    171
PpMFTL4           --------------------------------LIAPSKSCGRELVPYMGPRPPVGIHRYI    139
PpMFTL1           --------------------------------QATSTSSSGRELVPYIGPRPPIGIHRYI    139
PpMFTL2           --------------------------------QVTHTSSSGRELVPYMGPRPPIGIHRYA    139
SlMFT             -------------------------------------VGRGKEVLGYVGPRPPVGIHRYI    119
AtMFT             -------------------------------------PSRGKEILPYMEPRPPVGIHRYI    119
CcMFT-L3          ---------------------------------------------TIHGARPPVGIHRYI    105
CoMFT-L1          -------------------------------------PTQGKEILPYMGPRPPVGIHRYI    119
CcMFT-L1          -------------------------------------PTQGKEILPYMGPRPPVGIHRYI    119
TcMFT-L1          -------------------------------------PTRGKEILVYMGPRPPVGIHRYI    172
GaMFT-L1          -------------------------------------PTRGKEILAYMGPRPPVGIHRYI    119
GrMFT-L1          -------------------------------------PTRGKEILAYMGPRPPVGIHRYI    119
GhMFT-L1-Dt       -------------------------------------PTRGKEILAYMGPRPPVGIHRYI    119
GhMFT-L1-At       -------------------------------------PTRGKEILAYMGPRPPVGIHRYI    119
```

```
SlSP2G          -------------------------------------ASQGREMVEYMGPKPPAGIHRYV    125
CoMFT-L2        -------------------------------------ATKGRELVPYMGPCPPTGIHRYI    86
CcMFT-L2        -------------------------------------ATKGRELVPYMGPCPPTGIHRYI    124
TcMFT-L2        -------------------------------------ATKGKEMVPYMGPQPPTGIHRYI    121
GrMFT-L2        -------------------------------------ATKGRELVAYMGPQPPTGIHRYI    121
GhMFT-L2-Dt     -------------------------------------ATKGRELVAYMGPQPPTGIHRYI    121
GaMFT-L2        -------------------------------------ATKGRELVAYMGPQPPTGIHRYI    121
GhMFT-L2-At     -------------------------------------STKGRELVAYMGPQPPTGIHRYI    121
SlSP5G          -------------------------------------ATFGNEVVGYESPRPSMGIHRYI    118
SlSP11D         -------------------------------------VTFGNEVVSYECPRPTMGIHRLV    120
AtFT            -------------------------------------TTFGNEIVCYENPSPTAGIHRVV    121
AtTSF           -------------------------------------NAFGNEVVCYESPRPPSGIHRIV    121
SpSP11C         -------------------------------------VTFGNEVICYESPRPSMGIHRIV    122
SpSP6A          -------------------------------------TRFGNEIVCYENPTPTMGIHRFV    119
SlSP3D          -------------------------------------SSFGQEIVSYESPRPSMGIHRFV    120
CoSFT           -------------------------------------ATFGQEVVCYESPRPTVGIHRFA    120
CcSFT           -------------------------------------ATFGQEVVCYESPRPTVGIHRFT    120
TcSFT           -------------------------------------ASFGQEVVCYESPRPTVGIHRFL    120
GaSFT           -------------------------------------ASFGQEVVCYESPRPTVGIHRFV    120
GhSFT-At        -------------------------------------ASFGQEVVCYESPRPTVGIHRFV    120
GrSFT           -------------------------------------ASFGQEVVCYESPRPTVGIHRFV    120
GhSFT-Dt        -------------------------------------ASFGQEVVCYESPRPTVGIHRFV    120
SlSP            -------------------------------------CSFGREVVGYEMPRPNIGIHRFV    122
AtCEN           -------------------------------------VSFGKEIIGYEMPRPNIGIHRFV    122
```

```
CoSP              -------------------------------------ASFGREVVNYEMPRPNIGIHRFV     107
CcSP              -------------------------------------ASFGREVVNYEMPRPNIGIHRFV     107
TcSP              -------------------------------------ATFGREVVNYEMPRPNIGIHRFV     121
GrSP              -------------------------------------ATFGREMVNYEMPRPNIGIHRFV     121
GhSP-Dt           -------------------------------------ATFGREMVNYEMPRPNIGIHRFV     121
GaSP              -------------------------------------ATFGREMVNYEMPRPNIGIHRFV     121
GhSP-At           -------------------------------------ATFGREMVNYEMPRPNIGIHRFV     121
AtTFL1            -------------------------------------ATFGKEVVSYELPRPSIGIHRFV     124
SlSP9D            -------------------------------------ATFGRELVSYETPRPNIGIHRFV     120
GaTFL1-L2         -------------------------------------ATFGREVVNYEIPRPDIGIHRFV     117
GhTFL1-L2-At      -------------------------------------ATFGREVVNYEIPRPDIGIHRFV     117
GrTFL1-L2         -------------------------------------ATFGREVVNYEIPRPDIGIHRFV     117
GhTFL1-L2-Dt      -------------------------------------ATFGREVVNYEIPRPDIGIHRFV     117
GrTFL1-L1         -------------------------------------ATFGREVVSYENPKPNIGIHRFV     120
GhTFL1-L1-Dt      -------------------------------------ATFGREVVSYENPKPNIGIHRFV     120
GaTFL1-L1         -------------------------------------ATFGREVVSYENPKPNIGIHRFV     120
GhTFL1-L1-At      -------------------------------------ATFGREVVSYENPKPNIGIHRFV     120
TcTFL1            -------------------------------------ATFGREVVSYEIPRPNIGIHRFV     120
CoTFL1            -------------------------------------ITFGREVVSYEIPRPNIGIHRFV     121
CcTFL1            -------------------------------------ATFGREVVSYEIPRPNIGIHRFV     121
AtBFT             -------------------------------------ASFGREIVRYETPKPVAGIHRYV     121
SlBFT-L1          -------------------------------------ASFGREIISYVNPKPVIGIHRYV     122
SlBFT-L2          -------------------------------------VSFGKEIVSYESPKPVIGIHRYV     122
SlBFT-L3          -------------------------------------ISFGREIVCYETPKPVIGIHRYV     123
```

```
TcBFT          -------------------------------------ASFGREVVSYETPKPTVGIHRYV   133
GaBFT-L2       -------------------------------------VSFGRELISYEAPNPAVGIHRYV   121
GhBFT-L2-At    -------------------------------------VSFGRELVSYEAPNPAVGIHRYV   121
GrBFT-L2       -------------------------------------VSFGREVVSYETPNPAVGIHRYV   121
GhBFT-L2-Dt    -------------------------------------VSFGREVVSYETPNPAVGIHRYV   121
GhBFT-L1-Dt    -------------------------------------ASFGREVVSYETPKPTVGIHRYV   121
GrBFT-L1       -------------------------------------ASFGREVVSYETPKPTVGIHRYV   121
GaBFT-L1       -------------------------------------ASFGREVISYETPKPTVGIHRYV   121
GhBFT-L1-At    -------------------------------------ASFGREVISYETPKPTVGIHRYV   121
CoBFT          -------------------------------------ASFGREVVGYETPKPTVGIHRYV   122
CcBFT          -------------------------------------ASFGREVVGYETPKPIVGIHRYV   122
                                                                  *  ****

PpMFTL3        FVLFKQPTGK-PLLVTAPS---VRNNFNTRTFAVEHGLGFPVAATYFNAAKEPGSRRR--   225
PpMFTL4        FVLFRQPL-T-PFHITPPT---VRSNFNTRYFAAQCGLGLPVAATYLNAQKEPGSRRR--   192
PpMFTL1        FVLFKQPS-Q-SFLISPPA---ARNNFSTRNFAAYYGLGLPVAATYCNSQKEPASRNR--   192
PpMFTL2        FILFKQPS-T-PFLISPPT---VRNNFSTRNFASHYGLGLPVAATYCNAQKEPGSRRR--   192
SlMFT          LVLFRQNAPM-QEIFQAPV---ARAHFRTRMFAHQLDLGVPVATVYFNAHKEPANRKR*-   173
AtMFT          LVLFRQNSPV-GLMVQQPP---SRANFSTRMFAGHFDLGLPVATVYFNAQKEPASRRR--   173
CcMFT-L3       FVLFQQNGPM-GTAVQPPA---SRANFNTRLFADHLNLGLPVATVYINAQKEPISRRR--   159
CoMFT-L1       FVLFQQNGPM-GTAVQPPA---SRANFNTRLFADHLNLGLPVATVYFNAQKEPISRRR--   173
CcMFT-L1       FVLFQQNGPM-GTAVQPPA---SRANFNTRLFADHLNLGLPVATVYFNAQKEPISRRR--   173
TcMFT-L1       LVLFQQKGPL-GQ-VQQPA---SRANFSTRLFAQHLNLGQPVATVYFNAQKEPVSRRR*-   225
GaMFT-L1       LVLFQQKGPL-GA-VQQPA---TRANFSTRFFADHLNLGLPVATVYFNAQKEPVSRRR--   172
```

GrMFT-L1	LVLFQQKGPL-GA-VQQPA---TRANFSTRFFADHLNLGLPVATVYFNAQKEPVSRRR--	172
GhMFT-L1-Dt	LVLFQQKGPL-GA-VQQPA---TRANFSTRFFADHLNLGLPVATVYFNAQKEPVSRRR*-	172
GhMFT-L1-At	LVLFQQKGPL-GA-VQQPA---TRANFSTRFFADHLNLGLPVATVYFNAQKEPVSRRR*-	172
SlSP2G	FTLFRQKEAE-QVPHKPPQ---GRSNFKTRQFASDNGLDLPVAALYFNSQKEHAAHH---	178
CoMFT-L2	LALFKQERAAAAGGIQLPN---GRANFNTRQFAAQNGLGLPVAALYFNSHKEPALKKR--	141
CcMFT-L2	LALFKQGRAAEAGGIQLPN---GRANFNTRQFAAQNGLGLPVAALYFNSQKEPALKKR--	179
TcMFT-L2	LVLFKQERAT-EGGCQLPD---ARANFSTRQFAAQNSLGLPVAAVYFNSQKEPAVKKR*-	175
GrMFT-L2	LALFKQEGAM-EGRIQVAD---ARANFSTRRFAAQSRLGLPVAAVYFNSQKEPAAKKR--	175
GhMFT-L2-Dt	LALFKQEGAM-EGRIQVAD---ARANFSTRRFAAQNRLGIPVAAVYFNSQKEPAAKKR*-	175
GaMFT-L2	LALFKQEGAM-EGRIQVAD---ARANFSTRRFAAQNRLGLPVAAVYFNSQKEPAAKKR--	175
GhMFT-L2-At	LALFKQEGAM-EGRIQVAD---ARANFSTRRFAAQNRLGLPVAAVYFNSQKEPAAKKR*-	175
SlSP5G	FVLYRQ-LGC-DAID-APDIIDSRQNFNTRDFARFHNLGLPVAAVYFNCNREGGTGGRRL	175
SlSP11D	LVLFRQ-LRR-EIIY-APE---NRQNFDTREFAKLYNFGLPVAAVYFNCQRENGTGGRRI	174
AtFT	FILFRQ-LGR-QTVY-APG---WRQNFNTREFAEIYNLGLPVAAVFYNCQRESGCGGRRL	175
AtTSF	LVLFRQ-LGR-QTVY-APG---WRQQFNTREFAEIYNLGLPVAASYFNCQRENGCGGRRT	175
SpSP11C	FSLFRQ-LGR-ETVY-APN---WRQNFNTRQFAELYNLGLPVAAVYFNCQRENGTGGRRC	176
SpSP6A	LVLFRQ-LGR-ETVY-PPG---WRQNF---------------------------------	140
SlSP3D	FVLFRQ-LGR-QTVY-APG---WRQNFNTRDFAELYNLGLPVAAVYFNCQRESGSGGRRR	174
CoSFT	FVLFRQ-LGR-QTVY-APG---WRQNFNTRDFAELYNLGLPVAAVYFNCQRESGSGGRRR	174
CcSFT	FVLFRQ-LGR-QTVY-APG---WRQNFNTRDFAELYNLGLPVAAVYFNCQRESGSGGRRR	174
TcSFT	FVLFRQ-LGR-QTVY-APG---WRQNFNTRDFAELYNLGLPVAAVYFNCQRESGSGGRRR	174
GaSFT	FVLFRQ-LGR-QTVY-APG---WRQNFNTRDFAELYNLGLPVAAVYFNCQRESGSGGRRT	174
GhSFT-At	FVLFRQ-LGR-QTVY-APG---WRQNFNTRDFAELYNLGLPVAAVYFNCQRESGSGGRRT	174
GrSFT	FVLFRQ-LGR-QTVY-APG---WRQNFNTRDFAELYNLGLPVAAVYFNCQRESGSGGRRT	174

GhSFT-Dt	FVLFRQ-LGR-QTVY-APG---WRQNFNTRDFAELYNLGLPVAAVYFNCQRESGSGGRRT	174
SlSP	FLLFKQ-KKR-QTISSAPV---SRDQFSSRKFSEENELGSPVAAVFFNCQRETAARRR--	175
AtCEN	YLLFKQ-TRR-GSVVSVPS---YRDQFNTREFAHENDLGLPVAAVFFNCQRETAARRR--	175
CoSP	FLLFKQ-KRR-QTVRFIPT---SRDQFNTRKFAEDNELGLPVAAVYFNAQRETAARRR--	160
CcSP	FLLFKQ-KRR-QTVRFTPT---SRDQFNTRKFAEDNELGLPVAAVYFNAQRETAARRR--	160
TcSP	FLLFKQ-KRR-QAVISTPS---SRDHFNTRKFAEENELGLPVAAVYFNAQRETAARRR*-	174
GrSP	FLLFKQ-KGR-QTVRSIPS---SRDRFYTRKFAEENELGVPVAAVYFNAQRETAARRR--	174
GhSP-Dt	FLLFKQ-KGR-QTVRSIPS---SRDRFDTRKFAEENELGVPVAAVYFNAQRETAARRR*-	174
GaSP	FLLFKQ-KGR-QTVRSIPS---SRDRFDTRKFAEENELGVPVAAVYFNAQRETAARRR--	174
GhSP-At	FLLFKQ-KGR-QTVRSIPS---SRDRFDTRKFAEENELGVPVAAVYFNAQRETAARRR*-	174
AtTFL1	FVLFRQ-KQR-RVIFPNIP---SRDHFNTRKFAVEYDLGLPVAAVFFNAQRETAARKR--	177
SlSP9D	FVLFKQ-KSR-SSVS-QPT---SRDHFNTRNFAQENNLEQPVTAVFFNAQRETAARRR--	172
GaTFL1-L2	FVLFKQ-KRR-QVIR-SPS---SRDNFNTRDFAAENDLDLPVAAVYFNARRETAARRR--	169
GhTFL1-L2-At	FVLFKQ-KRR-QVIR-SPS---SRDNFNTRDFAAENDLDLPVAAVYFNARRETAARRR*-	169
GrTFL1-L2	FVLFKQ-KRR-QVIR-SPS---SRDNFNTRDFAAENDLGLPVAAVYFNARRETAARRR--	169
GhTFL1-L2-Dt	FVLFKQ-KRR-QVIR-SPS---SRDNFNTRDFAAENDLGLPVAAVYFNARRETAARRR*-	169
GrTFL1-L1	FVLFKQ-KRR-QIIK-SPC---SRDNFNTRRFASENDLGLPVAAVYFNAQRETAARRR--	172
GhTFL1-L1-Dt	FVLFKQ-KRR-QIIK-SPC---SRDNFNTRRFASENDLGLPVAAVYFNAQRETAARRR*-	172
GaTFL1-L1	FVLFKQ-KRR-QIIK-SPC---SRDNFNTRRFAFENDLGLPVAAVYFNAQRETAARRR--	172
GhTFL1-L1-At	FVLFKQ-KRR-QIIK-SPC---SRDNFNTRRFASENDLGLPVAAVYFNAQRETAARRR*-	172
TcTFL1	FVLFKQ-KRR-QMIT-SPS---SRDNFSTRGFAAENDLGLPVAAVYFNAQRETAARRR*-	172
CoTFL1	FVLFKQ-KRR-QIIK-PPS---SRDNFSTRDFAAENDLGLPVAAVYFNAQRETAARRR--	173
CcTFL1	FVLFKQ-KRR-QIIK-PPS---SRDNFSTRDFAAENDLGLPVAAVYFNAQRETAARRR--	173
AtBFT	FALFKQ-RGR-QAVKAAPE---TRECFNTNAFSSYFGLSQPVAAVYFNAQRETAPRRRPS	176

```
SlBFT-L1         FVLYKQNRGR-QT-VKPSV---SRDHFNTRKFAVENGLGSPVAAVYFNAQRETAARRR*-     175
SlBFT-L2         FILYKQNRGR-QT-VKPPV---TRDHFNARKFAVENGLGSPVAAVYFNAQRETAARRR*-     175
SlBFT-L3         FLLYKQ-RGR-QT-VRAPA---TRDQFNTRSFSAENGLGSPVAAVYFNAQRETAARRR*-     175
TcBFT            FILFKQ-RGR-QT-VRPPT---SRDYFNTRRFSQENGLGLPVAAVYFNAQRETAARRR*-     185
GaBFT-L2         FILFKQ-RGR-RT-VKSPS---SRDYFNTRRFSAENGLGLPVAAVYFNAQRETAARRR--     173
GhBFT-L2-At      FILFKQ-RGR-RT-VKSPS---SRDYFNTRRFSAENGLGLPVAAVYFNAQRETAARRR*-     173
GrBFT-L2         FILFKQ-RGR-RT-VKSPS---SRDYFNTRRFSAENGLGLPVAAVYFNAQRETAARRR--     173
GhBFT-L2-Dt      FILFKQ-RGR-RT-VKSPS---SRDYFNTRRFSAENGLGLPVAAVYFNAQRETAARRR*-     173
GhBFT-L1-Dt      FVLFKQ-RGR-KT-VRPPS---SRDCFNTRRFSADNGLGLPVAAVYFNAQRETAARSRR*     174
GrBFT-L1         FVLFKQ-RGR-QT-VRPPS---SRDCFNTRRFSADNGLGLPVAAVYFNAQRETAARSRR-     174
GaBFT-L1         FVLFKQ-RGR-QT-VRPPS---SRDCFNTRRFSADNGLGLPVAAVYFNAQRETAARSRR-     174
GhBFT-L1-At      FVLFKQ-RGR-QT-VRPPS---SRDCFNTRRFSADNGLGLPVAAVYFNAQRETAARSRR*     174
CoBFT            FVLFKQ-RGR-QT-VRPPS---SRDYFNTRSFSEENGLGLPVAAVYFNAQRETAARRR--     174
CcBFT            FVLFKQ-RGR-QT-VRPPS---SRDYFNTRSFSEENGLGLPVAAVYFNAQRETAARRR--     174
                   *::*                 *  *

PpMFTL3          ---    225
PpMFTL4          ---    192
PpMFTL1          ---    192
PpMFTL2          ---    192
SlMFT            ---    173
AtMFT            ---    173
CcMFT-L3         ---    159
CoMFT-L1         ---    173
```

CcMFT-L1	---	173
TcMFT-L1	---	225
GaMFT-L1	---	172
GrMFT-L1	---	172
GhMFT-L1-Dt	---	172
GhMFT-L1-At	---	172
SlSP2G	---	178
CoMFT-L2	---	141
CcMFT-L2	---	179
TcMFT-L2	---	175
GrMFT-L2	---	175
GhMFT-L2-Dt	---	175
GaMFT-L2	---	175
GhMFT-L2-At	---	175
SlSP5G	---	175
SlSP11D	*--	174
AtFT	---	175
AtTSF	---	175
SpSP11C	E*-	177
SpSP6A	---	140
SlSP3D	SAD	177
CoSFT	---	174
CcSFT	---	174
TcSFT	*--	174

GaSFT	---	174
GhSFT-At	*--	174
GrSFT	---	174
GhSFT-Dt	*--	174
SlSP	---	175
AtCEN	---	175
CoSP	---	160
CcSP	---	160
TcSP	---	174
GrSP	---	174
GhSP-Dt	---	174
GaSP	---	174
GhSP-At	---	174
AtTFL1	---	177
SlSP9D	---	172
GaTFL1-L2	---	169
GhTFL1-L2-At	---	169
GrTFL1-L2	---	169
GhTFL1-L2-Dt	---	169
GrTFL1-L1	---	172
GhTFL1-L1-Dt	---	172
GaTFL1-L1	---	172
GhTFL1-L1-At	---	172
TcTFL1	---	172

```
CoTFL1            ---    173
CcTFL1            ---    173
AtBFT             Y--    177
SlBFT-L1          ---    175
SlBFT-L2          ---    175
SlBFT-L3          ---    175
TcBFT             ---    185
GaBFT-L2          ---    173
GhBFT-L2-At       ---    173
GrBFT-L2          ---    173
GhBFT-L2-Dt       ---    173
GhBFT-L1-Dt       ---    174
GrBFT-L1          ---    174
GaBFT-L1          ---    174
GhBFT-L1-At       ---    174
CoBFT             ---    174
CcBFT             ---    174
```

Supplementary Figure S1: CETS polypeptide alignment. *G raimondii* (Gr), *G. arboreum* (Ga), and *G. hirsutum* (Gh, NAU-NBI) CETS protein sequences are aligned with CETS from Arabidopsis (*Arabidopsis thaliana*, At), jute (*Corchorus olitorius* and *Corchorus capsularis*, Co and Cc), cacao (*Theobroma cacao*, Tc), tomato (*Solanum lycopersicum* and *Solanum pimpinellifolium*, Sl and Sp), and moss (*Physcomitrella patens*, Pp) using Clustal Omega. "***"** indicates identical residues, "**:**" indicates conserved residues (scoring > 0.5 in the Gonnet PAM 250 matrix), and "**.**" denotes conservation between amino acids with weakly similar properties (scoring < 0.5 in the Gonnet PAM 250 matrix).

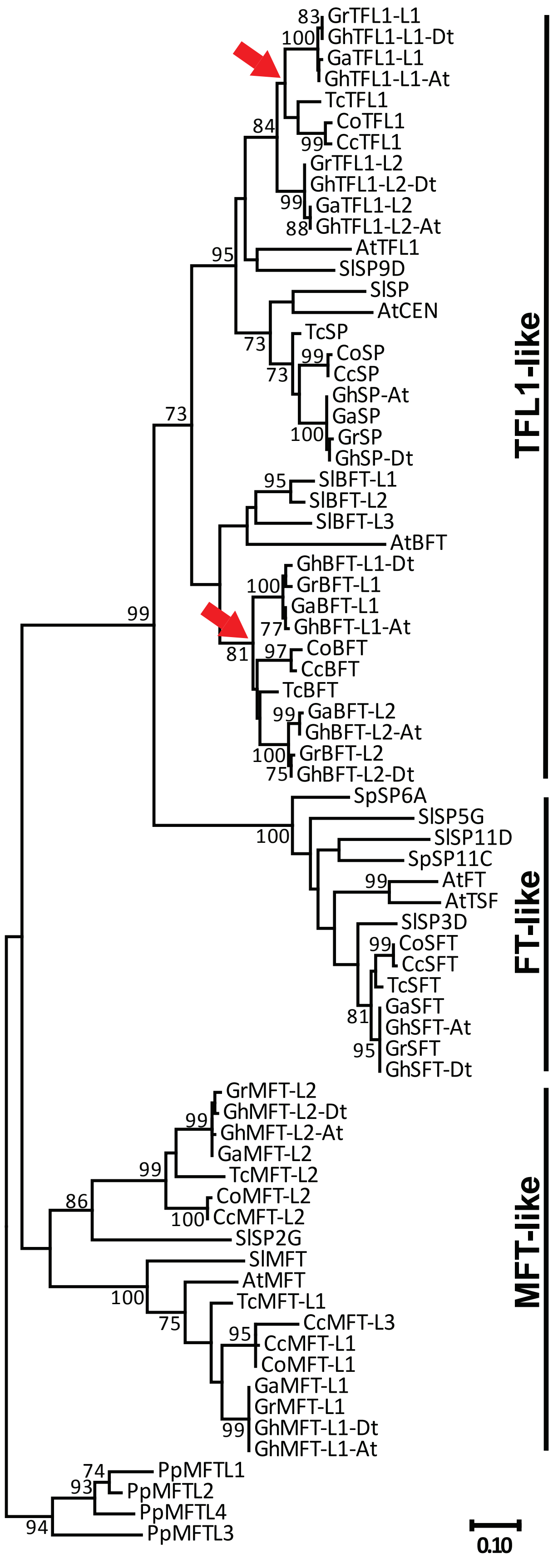

Supplementary Figure S2: Duplications in the cotton *CETS* gene family are not observed in closely related malvales. Shown is a phylogenetic tree constructed from the predicted polypeptide sequences of the *G. raimondii* (Gr), *G. arboreum* (Ga), and *G. hirsutum* (Gh, NAU-NBI assembly) homologs with Arabidopsis (*Arabidopsis thaliana*, At*),* tomato (*Solanum lycopersicum*, Sl), cacao (*Theobroma cacao*, Tc), jute (*Corchorus capsularis* and *Corchorus olitorius*, Cc and Co), and moss (*Physcomitrella patens*, Pp) CETS. Red arrows point to gene duplications in *Gossypium*. The scale bar represents amino acid substitution frequency determined by the Poisson correction method. Evolutionary analyses were conducted in MEGA7. Tree branches are labeled with percentages of 1000 iterations support, and the tree is drawn to scale with branch lengths measured in the number of substitutions per site.

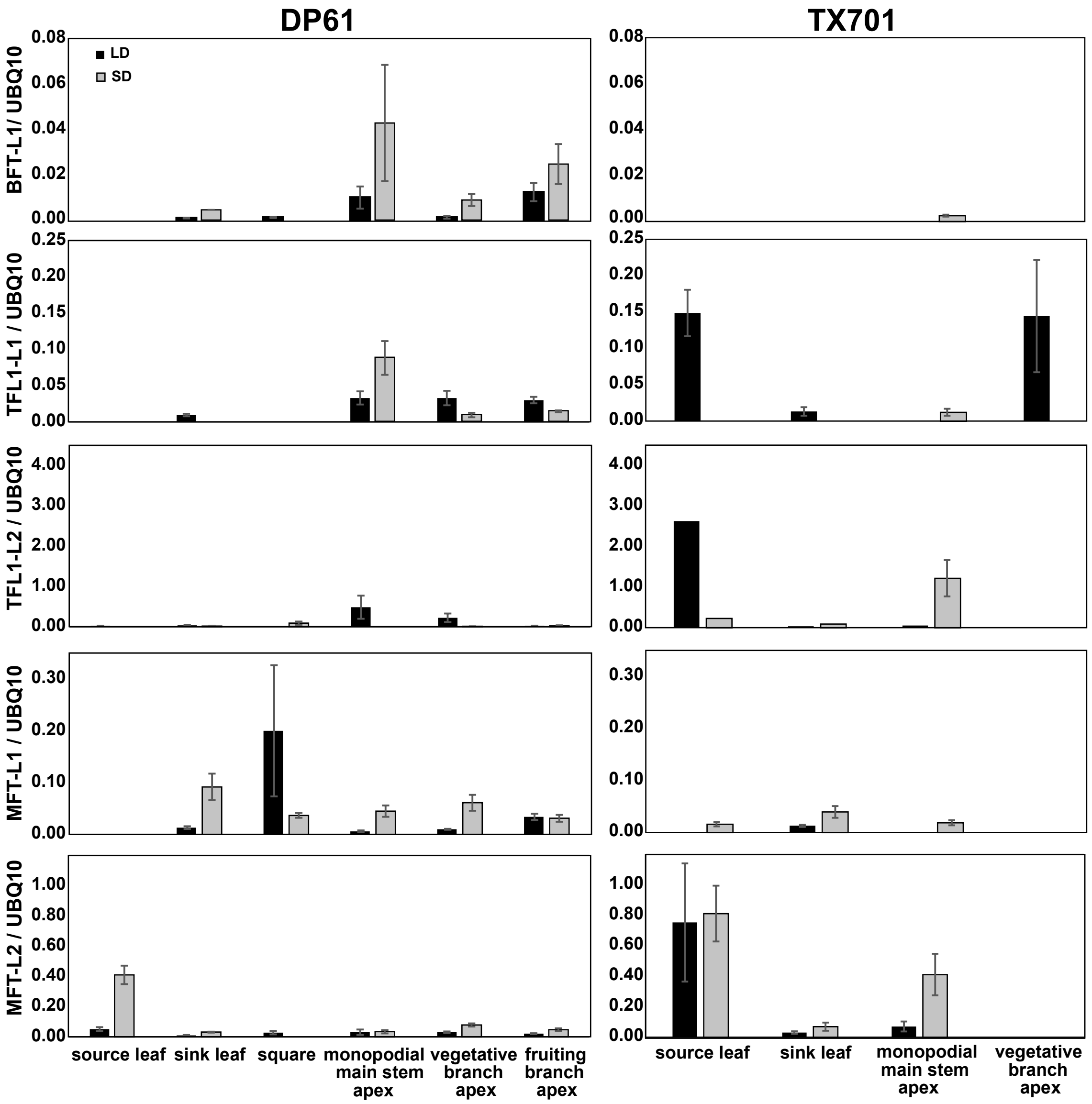

Supplementary Figure S3: Spatial expression profiles of *CETS* genes in cotton. The relative expression of *GhBFT-L1*, *GhTFL1-L1*, *GhTFL1-L2*, *GhMFT-L1*, and *GhMFT-L2* in the shoots of day-neutral DP61 and photoperiodic TX701 plants grown under inductive short-days (SD) or non-inductive long days (LD) was quantified by RT-qPCR. Expression was determined in source leaves, newly emerged sink leaves, immature floral buds ("square"), the monopodial main stem apex, the vegetative branch apex, and a reproductive fruiting branch apex, and compared with expression of *GhUBQ10* in these tissues.

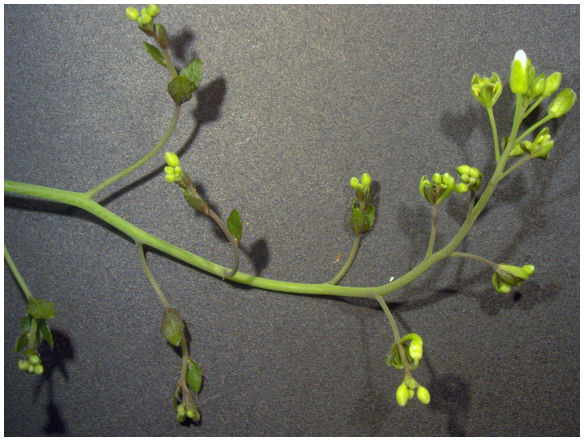

Supplementary Figure S4: Ectopic expression of genes from the *GhTFL1*-like clade produces the I1* phase, with nodes becoming increasingly flower-like. Shown in this *35S$_{pro}$:GhBFT-L1* plant, the basal nodes appear branch-like, and bear a few leaves before terminating with a floral cluster. Progressing distally along the inflorescence: nodes no longer have leaves; floral clusters arise from sepal-like structures; floral clusters arise from unfused carpels; and finally at the plant apex, normal flowers form.

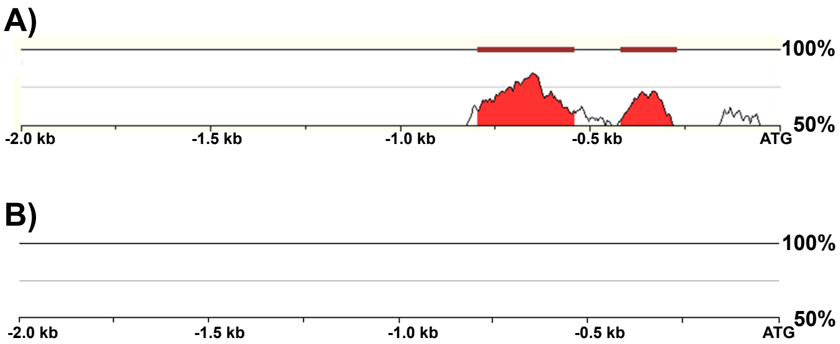

Supplementary Figure S5: Comparative promoter analysis of the *GhTFL1* and *GhBFT* paralogs. Shown are R-vista alignments of the 2 kb promoters from **(A)** *GhTFL1-L1* (Gohir.D09G135900) and *GhTFL1-L2* (Gohir.D04G100700), and **(B)** *GhBFT-L1* (Gohir.D08G112000) and *GhBFT-L2* (Gohir.D11G009200). Evolutionarily conserved non-coding sequences shared between the paralogs are shown as red peaks.
